# Single-cell RNA-seq uncovers dynamic processes and critical regulators in mouse spermatogenesis

**DOI:** 10.1038/s41422-018-0074-y

**Published:** 2018-07-30

**Authors:** Yao Chen, Yuxuan Zheng, Yun Gao, Zhen Lin, Suming Yang, Tongtong Wang, Qiu Wang, Nannan Xie, Rong Hua, Mingxi Liu, Jiahao Sha, Michael D. Griswold, Jinsong Li, Fuchou Tang, Ming-Han Tong

**Affiliations:** 1 State Key Laboratory of Molecular Biology, Shanghai Key Laboratory of Molecular Andrology, CAS Center for Excellence in Molecular Cell Science, Shanghai Institute of Biochemistry and Cell Biology, Chinese Academy of Sciences, University of Chinese Academy of Sciences, Shanghai, 200031 China; 2 Beijing Advanced Innovation Center for Genomics, Biomedical Institute for Pioneering Investigation via Convergence, College of Life Sciences, Peking University, Beijing, 100871 China; 3 Ministry of Education Key Laboratory of Cell Proliferation and Differentiation, Beijing, 100871 China; 4 Peking-Tsinghua Center for Life Sciences, Peking University, Beijing, 100871 China; 5 State Key Laboratory of Reproductive Medicine, Nanjing Medical University, Nanjing, Jiangsu 211166 China; 6 School of Molecular Biosciences and the Center for Reproductive Biology, Washington State University, Pullman, WA USA

## Abstract

A systematic interrogation of male germ cells is key to complete understanding of molecular mechanisms governing spermatogenesis and the development of new strategies for infertility therapies and male contraception. Here we develop an approach to purify all types of homogeneous spermatogenic cells by combining transgenic labeling and synchronization of the cycle of the seminiferous epithelium, and subsequent single-cell RNA-sequencing. We reveal extensive and previously uncharacterized dynamic processes and molecular signatures in gene expression, as well as specific patterns of alternative splicing, and novel regulators for specific stages of male germ cell development. Our transcriptomics analyses led us to discover discriminative markers for isolating round spermatids at specific stages, and different embryo developmental potentials between early and late stage spermatids, providing evidence that maturation of round spermatids impacts on embryo development. This work provides valuable insights into mammalian spermatogenesis, and a comprehensive resource for future studies towards the complete elucidation of gametogenesis.

## Introduction

Mammalian spermatogenesis is a complex, asynchronous process during which diploid spermatogonia generate haploid spermatozoa. It proceeds through a well-defined order of mitotic expansions, meiotic reduction divisions, and spermiogenesis.^1,2^ A single (As) spermatogonia, which function as actual spermatogonial stem cells (SSCs), either self-renew or divide into A-paired (Ap) spermatogonia. Ap then produce A-aligned (Aal) spermatogonia, which differentiate into type A1 spermatogonia without a mitotic division and then undergo a series of mitotic divisions to further generate successive types A2, A3, A4, intermediate (In), and B spermatogonia. As, Ap, and Aal are termed “undifferentiated spermatogonia”, whereas types A1 to B spermatogonia are termed “differentiating spermatogonia”.^3^ The type B spermatogonia give rise to preleptotene spermatocytes, which undergo a prolonged S phase followed by a highly regulated meiotic prophase I. The most complex and critical events of spermatogenesis, including recombination and synapsis, take place in this meiotic prophase I, which is subdivided into four cytological stages: leptonema, zygonema, pachynema, and diplonema. After meiotic prophase I, spermatocytes undergo two rounds of chromosome segregation, resulting in the production of haploid round spermatids. Subsequently, these round spermatids undergo dramatic morphological and biochemical changes to form elongated mature spermatozoa. This process is termed spermiogenesis. Mouse spermatids ranging from round to elongated cells can be morphologically defined as steps 1–8 round spermatids, and steps 9–16 elongating spermatids.^2^

All of these steps require the coordinated interaction of multiple molecules, whose expression is precisely controlled in time and space.^4,5^ In recent years, genome-wide microarray and RNA-sequencing (RNA-seq) studies of enriched spermatogenic cell populations or testis samples from model animals have provided knowledge of the molecular control underlying mammalian spermatogenesis.^6–14^ However, asynchronous spermatogenesis and the lack of an effective in vitro system have hindered efforts to isolate highly homogeneous populations of stage-specific spermatogenic cells. This has precluded the molecular characterization of spermatogenic cells at defined stages, and thereby an understanding of the spatiotemporal dynamics of spermatogenesis, in particular cellular transitions, at the molecular level.

The most common approaches used to isolate spermatogenic cells include fluorescence-activated cell sorting (FACS) and STA-PUT.^15^ However, they only allow separation of limited subtypes of enriched male germ cells. The major challenge remains isolating high-purity homogeneous spermatogenic cells of all subtypes from mouse testis. Isolation specifically of type B spermatogonia, for example, which represents the last mitotic cells before entry into meiotic prophase, and G1 and S phase preleptotene spermatocytes, could elucidate the mitotic-to-meiotic switch in mammals. However, the lack of specific markers for distinguishing differentiated spermatogonia (types A1 to B) has hampered their purification. In addition, although several alternative splicing (AS) studies during male germ cell development have been recently performed in mice, based on STAPUT-enriched spermatogenic cell populations (mainly spermatogonia, pachytene/diplotene spermatocytes, and round spermatids),^6,16,17^ they do not allow definitive assignment of specific AS events to a specific cell type or determination of the AS switch between neighboring stages such as occurring in mitotic-to-meiotic cells or meiotic-to-postmeiotic cells. Furthermore, the molecular identities and embryo developmental potentials of the multiple specialized subtypes of round spermatids are not fully understood because they have been defined by a combination of all subtypes. Thus, these limitations of cell purity have been particularly a problem to decipher the molecular hallmarks defining the premeiotic and meiotic spermatocytes, and the round spermatid sub-stages, and to elucidate the molecular basis of the mitotic-to-meiotic switch and the meiotic-to-postmeiotic transition in mammals. This leaves major gaps in our knowledge of the molecular mechanisms controlling spermatogenesis.

Single-cell RNA-seq is an unbiased approach that has extended our understanding of heterogeneous tissues, including mouse and human embryonic gonads.^18–20^ We reasoned that analysis of stage-specific gene expression profiles of individual spermatogenic cells could provide unbiased and novel insights into their molecular details. In this work, we developed a combinatorial method to purify all relevant types of mouse synchronous and homogenous spermatogenic cells. Applying single-cell RNA-seq with dense time points to these individual spermatogenic cells elucidated dynamic processes and functional characteristics, defined molecular events across male germ cell development, and revealed several novel crucial regulators of mammalian spermatogenesis.

## Results

### Strategy for uncovering dynamic processes and critical regulators of spermatogenesis

To gain insights into the molecular control of spermatogenesis, we devised an experimental strategy to: (i) establish mouse models carrying germ cell-specific markers; (ii) synchronize spermatogenesis in mice carrying these germ cell-specific fluorescent markers; (iii) isolate the synchronous spermatogenic cells at different developmental stages including mitotic, meiotic, and postmeiotic stages; (iv) validate the identity of the sorted cells using electron and light microscopy, as well as immunostaining for stage-specific and cell cycle-specific markers; (v) perform single-cell RNA-seq on spermatogenic cells; (vi) identify clusters of cells at stages that are similar to each other, and particular molecular signatures per cluster and cell type; (vii) perform intracytoplasmic round spermatid injection (ROSI) to determine the embryo developmental potentials of round spermatids at different stages; (viii) define specific molecular events such as alternative splicing (AS) and meiotic sex chromosome silencing (MSCI) during spermatogenesis; and (ix) perform functional analyses for selected molecular regulators.

### Establishing an approach to purify homogenous spermatogenic cells of all types from mice

**Fig. 1:**
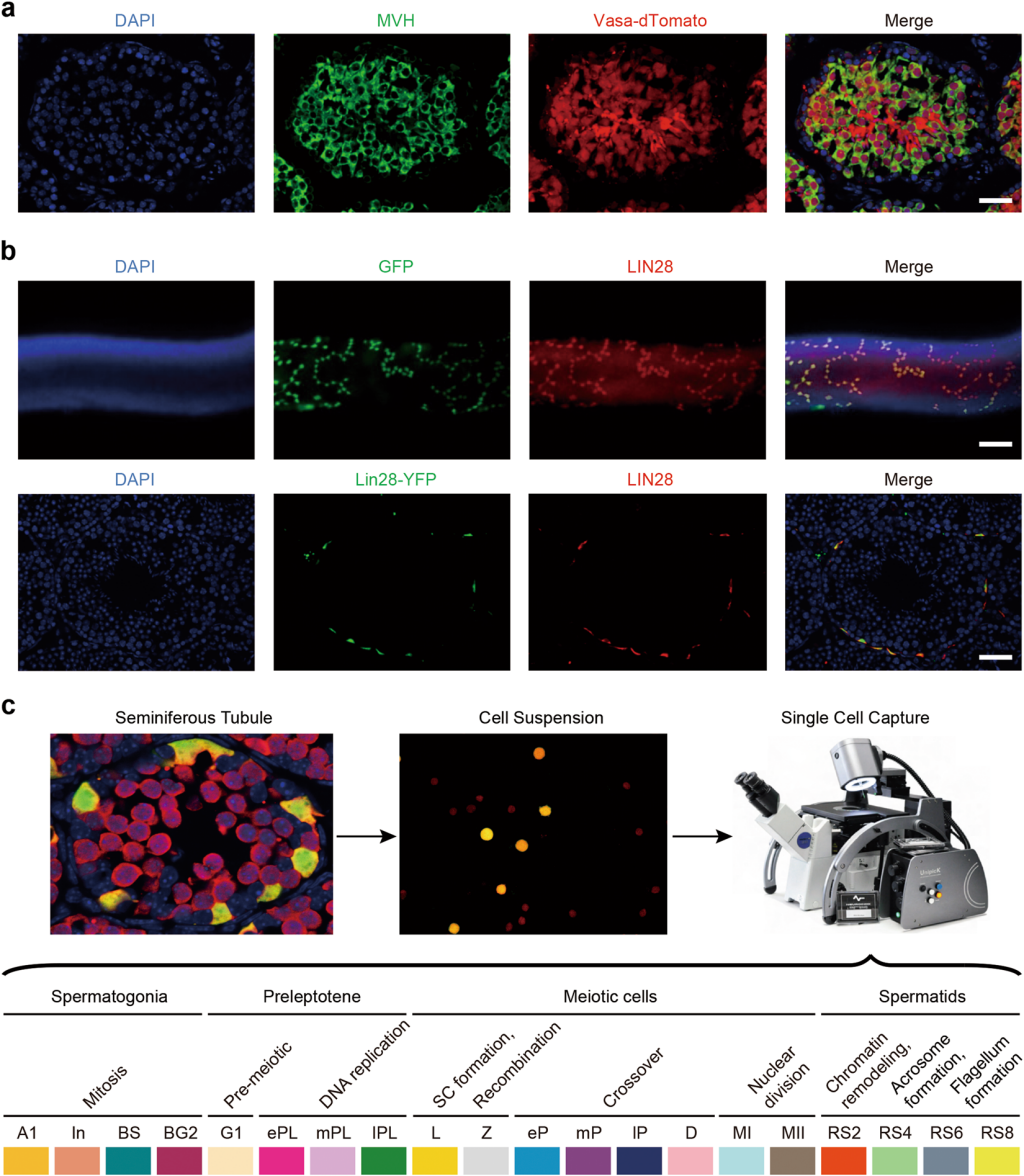
Isolation of all types of mouse spermatogenic cells. **a** Differential expression of transgene dTomato revealed by immunofluoresence in *Vasa*-dTomato knockin mice. Detection of the dTomato (red) signal costained with VASA (green) in *Vasa*-dTomato mice. Scale bar, 50 μm. **b** Differential YFP expression detected by immunofluoresence in *Lin28*-YFP knockin mice. Whole-mount immunostaining of seminiferous tubules for GFP (green) and LIN28 (red) (top panel) in *Lin28*-YFP mice. Detection of the YFP (green) signal costained with LIN28 (red) (bottom panel) in *Lin28*-YFP mice. Scale bar, 50 μm. **c** Schematic of the workflow. Male mice carrying both *Vasa*-dTomato and *Lin28*-YFP alleles were synchronized. Differential fluorescent protein expression as seen by immunofluorescence in undifferentiated spermatogonia (Green + Red = Orange) and preleptotene spermatocytes (Red) at the stage IV seminiferous tubule. Testes were dissociated into single-cell suspension, and sorted by FACS at population levels. The single cells were picked using the Unipick system according to fluorescence and cell size. In total, twenty subtypes of spermatogenic cells were profiled, including differentiated spermatognia (A1 type A1 spermatogonia, In intermediate spermatogonia, BS S phase type B spermatogonia, BG2 G2/M phase type B spermatogonia), preleptotene spermatocytes (G1 G1 phase preleptotene, ePL early S phase preleptotene, mPL middle S phase preleptotene, lPL late S phase preleptotene), meiotic cells (L leptotene, Z zygotene, eP early pachytene, mP middle pachytene, lP late pachytene, D diplotene, MI metaphase I, MII metaphase II), and round spermatids (RS2 steps 1–2 spermatids, RS4 steps 3–4 spermatids, RS6 steps 5–6 spermatids, RS8 steps 7–8 spermatids)

Our studies relied on four critical features: optimized synchronous spermatogenesis; dense time points that oversampled the mitotic-to-meiotic transition, meiotic development, meiotic-to-postmeiotic transition, and round spermatid differentiation; in-depth validation of staging at each time point; and single-cell RNA-seq. To purify homogenous spermatogenic cells, we first engineered *Vasa*-dTomato and *Lin28*-YFP knock-in mouse lines (Supplementary information, Fig. S1a). The spermatogenic cells in *Vasa*-dTomato mice express a red fluorescent protein dTomato marker, whereas the undifferentiated spermatogonia in *Lin28*-YFP mice express a yellow fluorescent protein (YFP) marker (Fig. 1a, b). Male mice carrying both *Vasa*-dTomato and *Lin28*-YFP alleles were then treated with WIN 18,446/retinoic acid (RA) to allow synchronization of spermatogenesis (Supplementary information, Fig. S1b, c).^21^ After spermatogenesis was synchronized, the spermatogenic cells in the testis included the undifferentiated spermatogonia that are labeled by both YFP and dTomato, and the synchronous advanced spermatogenic cells at specific stages (from type In spermatogonia onwards), which are only labeled by dTomato (Fig. 1c). In total, the testicular tissue samples at 20 time points were collected for subsequent experiments (Supplementary information, Fig. S1d, see Materials and Methods). At each time point post RA treatment, we performed transmission electron microscopy (TEM) and immunostaining for stage-specific and cell cycle-specific markers to determine the degree and seminiferous epithelial stage of synchronous spermatogenesis (Supplementary information, Figs. S2-19). We then isolated the spermatogenic cell populations by FACS, and individual spermatogenic cells using the Unipick system based on dTomato, GFP, DNA content, and cell size (Fig. 1c). Cytological studies revealed a high degree of synchrony and purity in isolated spermatogenic cells and provided a cytological framework to ensure the acquisition of accurate gene expression data (Supplementary information, Figs. S2-19). More importantly, the methods we established allowed us to obtain high-purity synchronous and homogeneous spermatogenic cells for any desired stage. For example, we isolated the G1 phase preleptotene spermatocytes, which are rarely described in the literature but could be critical for understanding the mitotic-to-meiotic transition (Supplementary information, Fig. S6).^22^ In addition, according to the distribution of As, Ap, and Aal spermatogonia in the seminiferous tubules of *Lin28*-YFP knock-in mouse lines (Fig. 1b), we were able to isolate individual As, Ap, and Aal spermatogonia in situ using the Unipick system. Thus, the methods presented here enabled us to successfully isolate all types of homogeneous male germ cells from mice, thereby being readily available for further investigations into the regulation of spermatogenesis.

### A comprehensive transcriptome landscape of mouse mitotic, meiotic, and postmeiotic cells

**Fig. 2:**
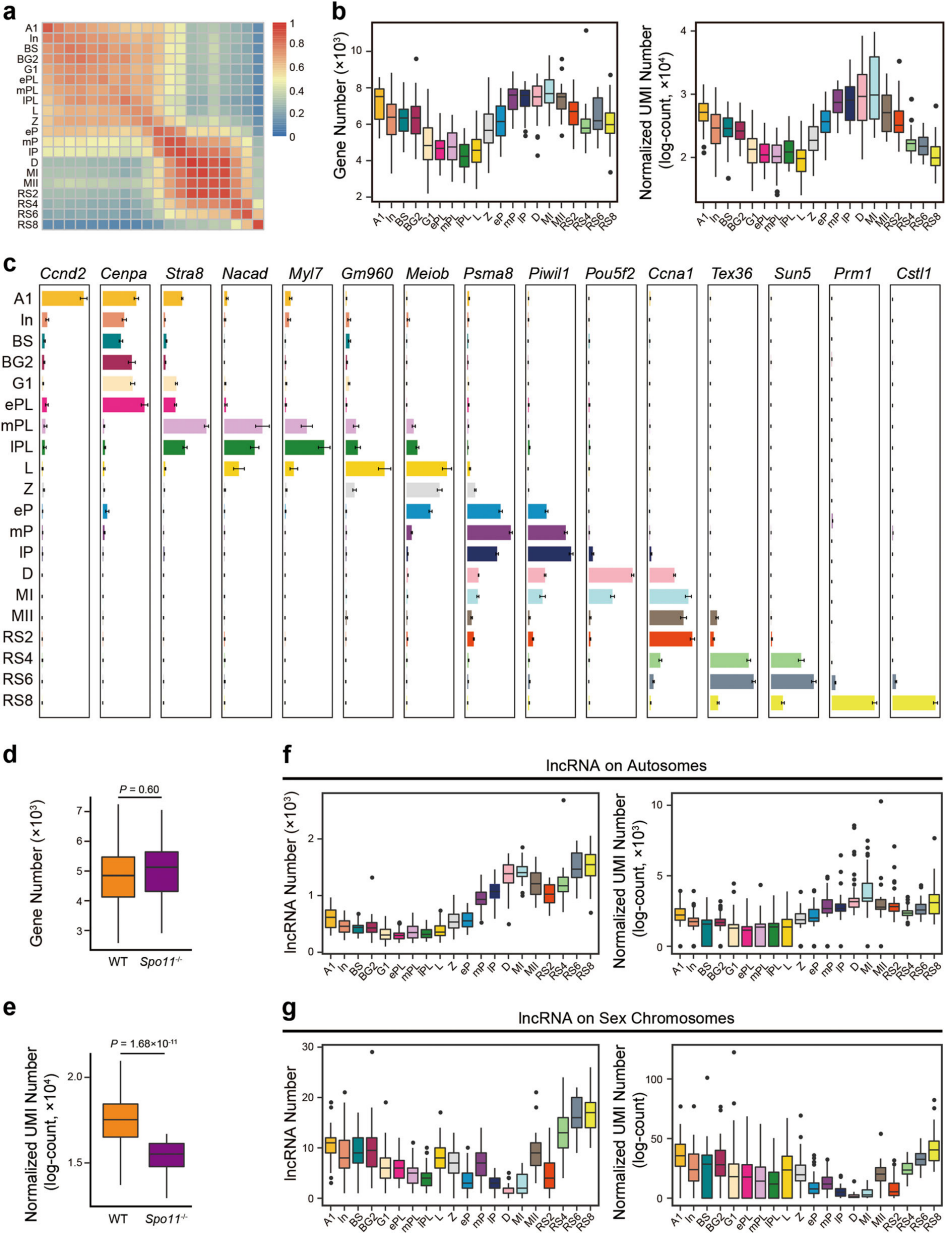
A comprehensive single-cell transcriptome atlas of mouse spermatogenesis. **a** Pearson correlation coefficient of all filtered spermatogenic cells between 20 developmental stages. The color key indicates the value of Pearson correlation coefficient from low (blue) to high (red). **b** Boxplots showing the gene number (left panel) and normalized UMI number (right panel) of known protein-coding genes expressed in each individual cell at different stages. Each boxplot represents the median, the first quartiles and the third quartiles of gene expression value; and the whiskers represent 1.5 times the interquartile range. The dots represent the outliers. **c** Bar plots showing the stage-specific representative gene expression based on RNA-seq analysis throughout 20 developmental stages. Error bar represents mean ± SEM. Scales of each gene expression are independent of each other. **d** Boxplots showing the gene numbers expressed in *Spo11*^−/−^ and wild-type (WT) control mice of leptotene stage based on single-cell RNA-seq. The *P* value is calculated by two-tailed Student’s *t*-test. **e** Boxplots showing the normalized UMI numbers expressed in *Spo11*^−/−^ and WT control mice of leptotene stage based on single-cell RNA-seq. The *P* value is calculated by two-tailed Student’s *t*-test. **f** Boxplots showing the numbers (left panel) and the normalized UMI numbers (right panel) of lncRNAs on autosomes expressed in each individual cell at 20 developmental stages. **g** Boxplots showing the numbers (left panel) and normalized UMI numbers (right panel) of lncRNAs on sex chromosomes expressed in each individual cell at 20 developmental stages

We profiled 1,204 individual cells from 20 developmental stages of synchronous spermatogenesis that comprehensively cover mitotic, meiotic, and postmeiotic stages, retaining 1,136 single-cell transcriptomes that passed stringent quality control (QC) filtering for subsequent analysis (Supplementary information, Fig. S20a and Table S1, see Materials and Methods). We observed an average of 290,537 copies of transcripts (UMIs) per spermatogenic cell, and detected an average of 6,367 uniquely expressed genes in each individual cell (transcripts per million (TPM) ≥ 1). Single-cell RNA-seq also revealed a high similarity of gene expression among individual spermatogenic cells at the same stage, demonstrating a high degree of spermatogenic cell synchrony and purity (Fig. 2a and Supplementary information, Table S1).

We found that the large majority of known protein-coding genes (18,037 out of 20,088, 89.8%) were transcribed in spermatogenic cells, and most of them displayed dynamic expression and temporal regulation (Fig. 2b, c). We found that the preleptotene and leptotene spermatocytes expressed the lowest number of protein-coding transcripts among all the analyzed spermatogenic cells, which is inconsistent with the observation that transcription in leptotene and zygotene spermatocytes is inert.^23^ This suggested that premeiotic DNA replication and double-strand break (DSB) formation could limit gene transcription globally (Fig. 2b). To test this hypothesis, we profiled 44 individual leptotene spermatocytes from synchronized *Spo11*^−/−^ mice, in which little or no DSB occurred,^24,25^ and retained 38 single-leptotene spermatocyte transcriptomes that passed QC filtering (Supplementary information, Table S1). Notably, although there was no significant difference (Student’s *t-*test, *P* value = 0.60) in the number of expressed genes between wild-type (WT) and *Spo11*^−/−^ leptotene spermatocytes, spike-in-normalized UMI count sharply decreased (Student’s *t-*test, *P* value = 1.68 × 10^−11^) in *Spo11*^−/−^ leptotene spermatocytes (Fig. 2d, e). Thus, DSB formation may not be the key limiting factor for transcription during leptonema.

We also identified 9,431 (78.8%) out of 11,962 annotated long noncoding RNAs (lncRNAs) expressed in spermatogenic cells, with striking stage-specific expression patterns. The highest number and expression level of lncRNAs were found in the diplotene and MI spermatocytes, as well as steps 6–8 spermatids (Fig. 2f, g).

### Dynamic gene expression patterns reveal the molecular signatures during spermatogenesis

**Fig. 3:**
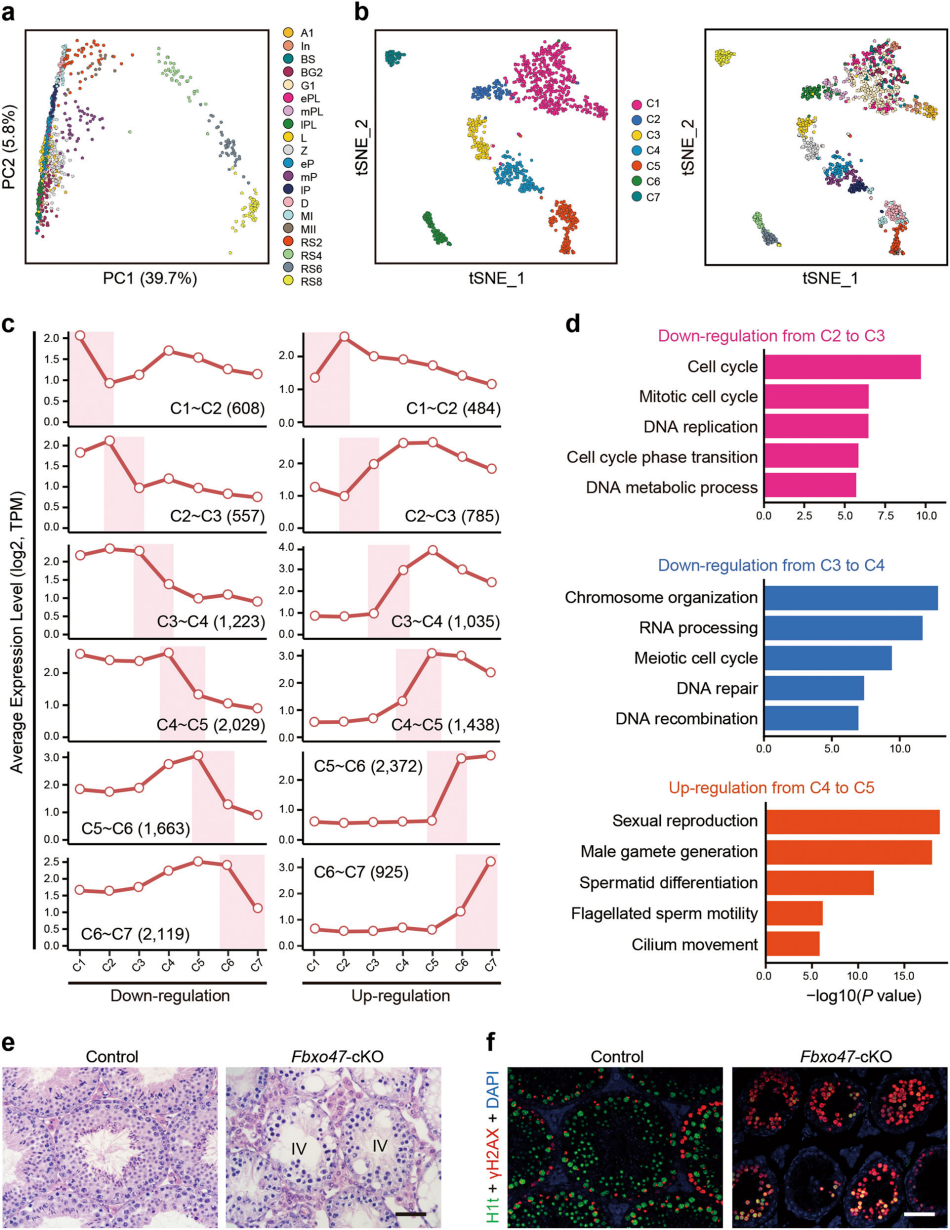
Characterization of dynamic gene expression patterns in male germ cell development. **a** Principal component analysis (PCA) of the spermatogenic cells at 20 different stages based on their gene expression pattern exhibited by PC1 and PC2. The variation values of PC1 and PC2 are 39.7 and 5.8%, respectively. Distinct cell types are shown in different colors. **b** The *t*-distribute stochastic neighbor embedding (*t*-SNE) plot with seven clusters of spermatogenic cells (left panel) and their corresponding developmental stages (right panel). Clusters C1 to C7 are shown in different colors. Cells at different developmental stages are shown in different colors as in **a**. **c** Line graph showing average gene expression level of down- (left panel) and up-regulated (right panel) differentially expressed genes (DEGs) when comparing each of two consecutive clusters. *y* axis, log_2_(TPM/10 + 1). The DEG number of each group is shown in brackets. **d** Gene ontology (GO) analysis of down-regulated DEGs from clusters C2 to C3 (upper panel), down-regulated DEGs from C3 to C4 (middle panel) and up-regulated DEGs from C4 to C5 (bottom panel), respectively. The dynamic gene expression patterns of these three groups are shown in **c**. **e** Hematoxylin and eosin (H & E) staining of wild-type control and *Fbxo47*-cKO testis sections at 8 weeks old. In mutant testes, seminiferous epithelium was arrested at stage IV. IV: stage IV. Scale bar, 50 μm. **f** Immunohistochemical staining for the mid-late pachytene spermatocyte marker histone variants H1t (green), γH2AX (red), and DAPI (blue) in sections of 8-week-old wild-type control and *Fbxo47*-cKO testes. Scale bar, 50 μm

Principal component analysis (PCA) of gene expression from spermatogenic cells at all 20 developmental stages together showed that the spermatogenic cells are accurately aligned along a developmental trajectory that most likely represents their path from a premeiotic state/meiotic prophase I (including spermatogonia, preleptotene spermatocytes, and prophase I spermatocytes), metaphase I and II states, and, finally to step 8 round spermatids, which is a postmeiotic state (Fig. 3a and Supplementary information, Fig. S20b). PC1 mainly reflected the gene expression changes between pre-round spermatid stages and round spermatids, whereas PC2 mainly reflected the changes from A1 spermatogonia to MII spermatocytes (Supplementary information, Fig. S20b). Moreover, the gene expression changes between the remaining cells could be discriminated by other PC dimensions. For example, PC6 revealed the gene expression changes between preleptotene, leptotene, and zygotene spermatocytes (data not shown). To classify the expression patterns into defined clusters, we then performed a *t*-distributed stochastic neighbor embedding (*t*-SNE) analysis with eleven significant PC dimensions not limited to PC1 and PC2 (see Materials and Methods). This revealed that the spermatogenic cells at 20 stages could be grouped into seven distinct main clusters, which reflected the global gene expression pattern, named cluster C1 to cluster C7 (Fig. 3b and Supplementary information, Table S2). We also investigated the batch effect of our data across these clusters, and found that cells captured from the same cell types were grouped into the same clusters, with no significant influence among different experiment batches (Supplementary information, Fig. S20c). We further analyzed these seven clusters and identified distinct characteristics (Supplementary information, Fig. S20d). In some cases, spermatogonic cells from different morphological stages were located in the same main cluster (Fig. 3b). For example, cluster C1 contained all of the mitotic cells (from types A1 to B spermatogonia) and premeiotic cells (from G1 phase to early S phase preleptotene spermatocytes), suggesting that the transcriptomes of mitotic spermatogonia are very similar to that of early preleptotene spermatocytes. The transcriptional profiles of leptotene and zygotene spermatocytes, where chromosome recombination and synaptonemal complex (SC) formation take place, were also relatively similar to each other and grouped into cluster C3. Interestingly, cluster C5 was predominantly composed of meiotic prophase I, meiotic metaphase, and postmeiotic cells, including diplotene, MI and MII spermatocytes, as well as steps 1–2 spermatids. Unexpectedly, the same type of spermatogenic cells sometimes fell into different clusters. For example, preleptotene spermatocytes fell into two apparently different clusters (clusters C1 and C2). This suggests that the mitotic-to-meiotic transcriptional switch could occur at the preleptotene stage. In particular, round spermatids exhibited a high degree of diversity and fell into three clusters (clusters C5-C7). Taken together, these results uncover previously unrecognized characteristics of spermatogenic cells, highlighting the necessity of spermatogenic synchrony, closely related time points, and single-cell technologies for dissecting spermatogenesis in molecular detail.

Our datasets allowed us to dissect the precise temporal regulation and the signature genes for each cluster and each cell stage, as detailed below. We found that 9,205 genes showed significant and meaningful changes between the consecutive clusters, whereas 9,299 genes exhibited marked differences between neighboring cell stages (Fig. 3c, d, Supplementary information, Fig. S20e and Table S3, see Materials and Methods). These results also verified the reliability of cell sampling.

#### Mitotic spermatogonia and early preleptotene spermatocytes

The transcriptomes of mitotic spermatogonia clustered together with early stage preleptotene spermatocytes. Therefore, we compared the transcriptomes between each of the neighboring cell stages across all the six developmental subtypes in cluster C1. We identified 689, 373, 133, 168, and 275 differentially expressed genes among the six subtypes of cells (Supplementary information, Table S4), and performed gene ontology (GO) analysis on these genes (Supplementary information, Fig. S20f and Table S4). In brief, highly expressed genes in type A1 spermatogonia were associated with gene silencing and chromosome organization such as *Piwi2*, *Ddx4*, and histone cluster 1 (*Hist1*),^26^ as previously reported. Moreover, we noticed that transcriptional profilings of type In spermatogonia closely resembled that of type B spermatogonia, whereas the transcriptome of type B spermatogonia was very similar to that of G1 phase preleptotene spermatocytes. However, we found 1,225 significantly differentially expressed genes between types A1 and B spermatogonia. Furthermore, there was 633 or 712 markedly differentially expressed genes between In spermatogonia and G1 or early S phase preleptotene spermatocytes, respectively (Supplementary information, Table S4). We thus propose that, as development proceeds, spermatogonia gradually obtain the competency of commitment to meiosis. Interestingly, we also found that the FGF (fibroblast growth factor) signaling pathway was significantly repressed in preleptotene spermatocytes compared to spermatogonia, suggesting that the suppression of FGF pathway could play a role in the mitotic-to-meiotic transition (Supplementary information, Fig. S21a).

#### Chromosome behavior during meiosis

Meiosis is a fundamental aspect of sexual reproduction that produces gametes with half the chromosome content of the original progenitor cells. During meiosis prophase I, meiotic chromosomes undergo a number of complex events, including meiotic recombination, homologous chromosome pairing and synapsis, to allow reductive chromosome segregation to occur. We found that genes involved in DNA replication were highly enriched in cluster C2, including S phase preleptotene spermatocytes, where this process occurs (Fig. 3d and Supplementary information, Table S3). Moreover, genes crucial for chromosome recombination were clearly enriched in cluster C3, a time precisely corresponding to recombination occurrence and SC formation (Fig. 3d). These genes included many of the previously characterized genes, which are known to be essential for DSB formation and DNA repair such as *Prdm9*, *Spo11*, *Gm960*, *Meiob*, *Dmc1*, and *Mcm8* (Supplementary information, Table S3).^27–30^

Importantly, some of the novel enriched genes showed similar expression patterns to *Prdm9*, *Gm960*, and *Meiob*. These included *Fbxo47*, *Pparg*, and *Ccnb3*, which may also be involved in these critical processes (Supplementary information, Fig. S21b). To test this hypothesis, we selected *Fbxo47*, which encodes an F-box domain-containing protein of unknown function that has been linked with specific cancers in humans.^31^ We generated a germ cell-specific *Fbxo47*-knockout mouse line by crossing *Fbxo47*-floxed mice with *Stra8*-*GFPCre* mice (Supplementary information, Fig. S21c).^32^ We found that male mice deficient in *Fbxo47* were completely infertile. Histological analyses clearly showed that mutant testes displayed spermatogenesis arrest at the level of meiotic prophase I spermatocytes in seminiferous epithelial stage IV, equivalent to wild-type middle-pachytene (mid-pachytene) spermatocytes, suggestive of arrest before mid-pachynema (Fig. 3e). Immunostaining for HIST1H1T (H1t), a marker of mid-late pachynema,^33^ showed little H1t labeling of *Fbxo47* mutant spermatocytes that also harbor an abnormal XY body (Fig. 3f), further suggestive of meiotic recombination defects. Altogether, these results demonstrate a novel role for *Fbxo47* in the regulation of meiotic prophase I, when recombination takes place. This demonstrates the potential value of our dataset as a rich resource of candidates that play novel roles in the regulation of meiosis.

Complete synapsis and XY body formation are characteristics of pachytene nuclei. Consistent with this, genes involved in XY body formation and repair of a subpopulation of DSBs for generating crossovers showed an expression peak during pachytene stages (Supplementary information, Fig. S21d-f; see Materials and Methods). Unexpectedly, not only the transcription of genes for recombination but also those for chromosome segregation and meiotic nuclear division, which actually occur after meiotic prophase I, was dramatically decreased at pachynema, and reduced to the lowest levels at diplonema compared to leptonema and zygonema (Supplementary information, Fig. S21g, h).

Of the significantly changed genes between early preleptotene and middle preleptotene (mid-preleptotene) spermatocytes, hundreds of upregulated genes were strongly enriched for meiosis-related terms, including most of the genes known to be associated with recombination, synapsis, and chromosome segregation (Supplementary information, Table S5). In contrast, the downregulated genes were highly enriched for mitosis-related terms. This reinforces the theory that the transcriptional program for mitotic-to-meiotic transition may be switched on at the preleptotene stage before meiotic prophase.

#### Spermatid morphogenesis

During spermiogenesis, spermatids form the chromatoid body (CB), develop an acrosome and a flagellum, and undergo chromatin remodeling, resulting in dramatic morphological and cytological changes.^34^ The transcriptional levels of genes involved in CB formation, machete development, cilium movement, and acrosome formation reached their peaks in clusters C5 and C6, and then began decreasing in cluster C7, including *Spag6*, *Ttc26*, *Gopc*, and *Vsp54* (Fig. 3d, and Supplementary information, Fig. S21i-l).^34^ It is of note that most of these spermiogenesis-associated genes start to be expressed from the early pachytene onward, long before the onset of postmeiotic processes for which they are required, suggesting that the transcriptional programs for the postmeiotic processes may be switched on at the pachynema stage as previously described.^8^

Overall, our studies defined extensive novel molecular signatures associated with stage-specific development of male germ cells, and revealed that: (i) the transcriptional program for the mitotic-to-meiotic transition might be turned on before early preleptonema, (ii) the expression pattern of enriched genes strongly matches the biological processes in which their products play roles, and (iii) the approaches that we have developed provide clues for identifying novel functions for hundreds of uncharacterized genes during spermatogenesis.

### Steps 1–2 round spermatids have a lower embryo developmental potential than steps 7–8 spermatids

**Fig. 4:**
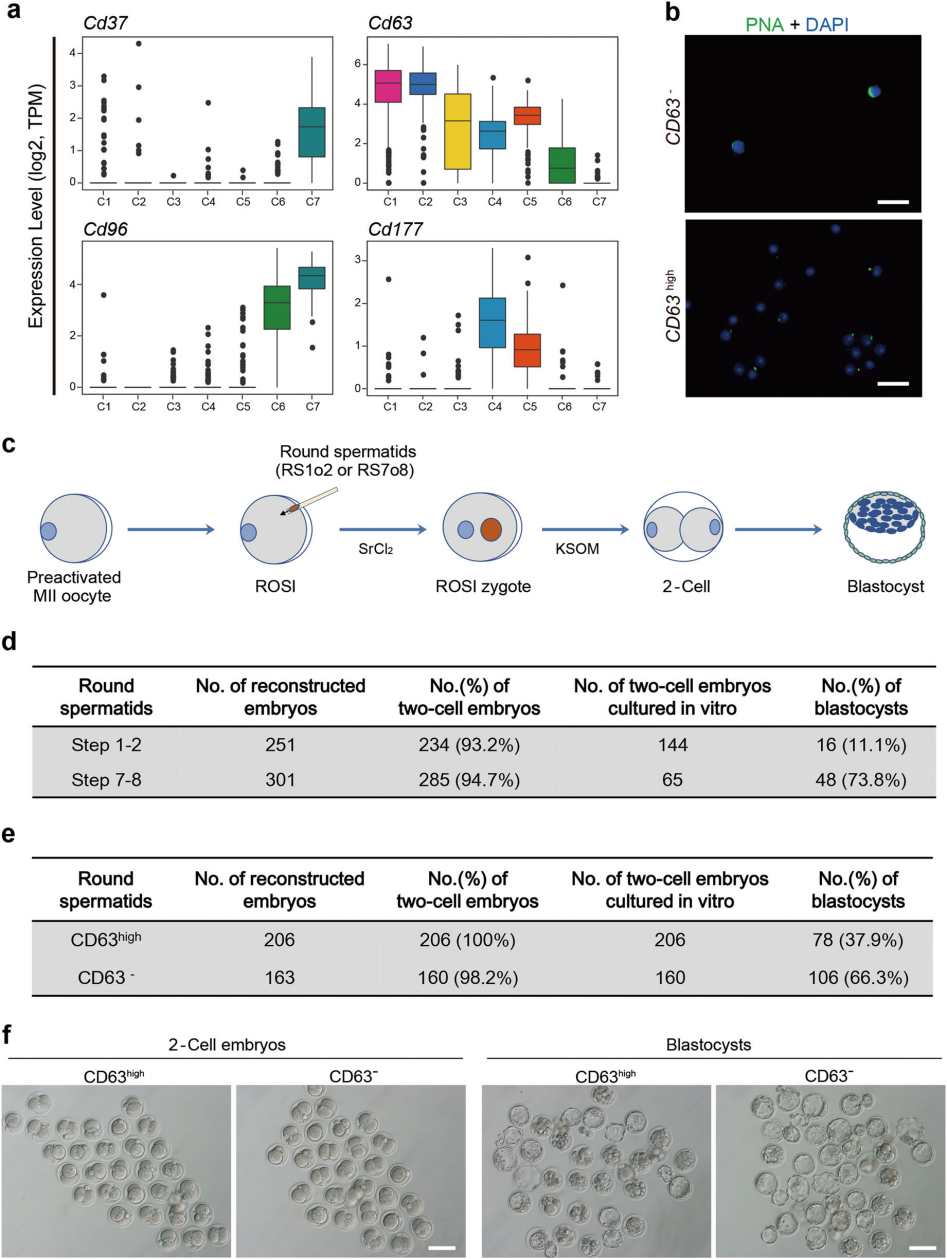
Steps 1-2 round spermatids have lower embryo developmental potential than Steps 7–8 spermatids. **a** Boxplots showing the expression levels of four representative surface markers, *Cd37* (upper left panel), *Cd63* (upper right panel), *Cd96* (bottom left panel) and *Cd177* (bottom right panel) detected in each individual cell in seven clusters defined in Fig. 3b. Expression levels were transformed to log_2_(TPM/10 + 1). **b** Immunocytochemical staining for acrosomal marker PNA (green) of isolated CD63^–^ (upper panel) and CD63^high^ (lower panel) round spermatids by FACS. Scale bar, 50 μm. **c** Schematic overview of intracytoplasmic round spermatid injection (ROSI) with steps 1–2 (RS1o2), and steps 7–8 (RS7o8) spermatids. **d** In vitro development of embryos injected with FACS-enriched steps 1–2 and steps 7–8 spermatids. **e** The efficiency of ROSI embryos developed to the blastocysts by injecting CD63^high^ and CD63^−^ round spermatids. **f** Images of 2-cell stage embryos and blastocysts generated by ROSI. Scale bar, 100 μm

Based on our single-cell dataset, we then screened cell surface markers that are specific to the different clusters of round spermatids (see Materials and Methods). We found that *Cd37*, *Cd63*, *Cd96*, and *Cd177* were differentially expressed in round spermatids (Fig. 4a). We then evaluated the efficacy of one candidate surface marker CD63 in distinguishing round spermatids at different stages, and found that the CD63^–^ subset in haploid cells was highly enriched with steps 7–8 spermatids, whereas the CD63^high^ population was enriched with steps 1–2 spermatids (Fig. 4b). This finding reveals that this set of discriminative markers could be used in combination to accurately isolate round spermatids at different developmental steps. This will enable further studies to elucidate the molecular mechanisms underlying spermatid differentiation.

Since round spermatids fell into three different clusters, we speculated that round spermatids at different stages may have different developmental potentials after injection into oocytes. To test this hypothesis, we first performed intracytoplasmic round spermatid injection (ROSI) using FACS-enriched spermatids synchronized at different stages (Fig. 4c). Oocytes, injected with spermatids from steps 1–2 and steps 7–8, were cleaved into two-cell embryos with an equivalent efficiency after activation (93.2 vs. 94.7%; Fig. 4d). However, the efficiency of acquiring expanded blastocysts from the two-cell embryos cultured in vitro, showed a significant difference between steps 1–2 and steps 7–8 spermatids. The higher efficiency was witnessed by injecting steps 7–8 spermatids. Of the 65 two-cell embryos, 48 developed to blastocysts at a rate of 73.8%, which is > 6-fold higher compared with that of steps 1–2 spermatids (11.1%; Fig. 4d). We then performed ROSI using CD63^–^ and CD63^high^ round spermatids, respectively, as described above. The efficiency of development to the blastocysts by injecting CD63^–^ round spermatids (enrichment of steps 7–8 spermatids) was significantly higher than that by injection of CD63^high^ round spermatids (enrichment of steps 1-2 spermatids), whereas progression to the two-cell embryos was not significantly different between two groups (Fig. 4e, f). Collectively, these findings reveal that late stage spermatids have a much higher embryo developmental potential, suggesting that suboptimal immature round spermatids may be responsible for the low success rate of clinical ROSI.^35,36^ Notably, the present study provides an approach to purify late stage round spermatids for ROSI in clinic.

### Transcriptional control during spermatogenesis

**Fig. 5:**
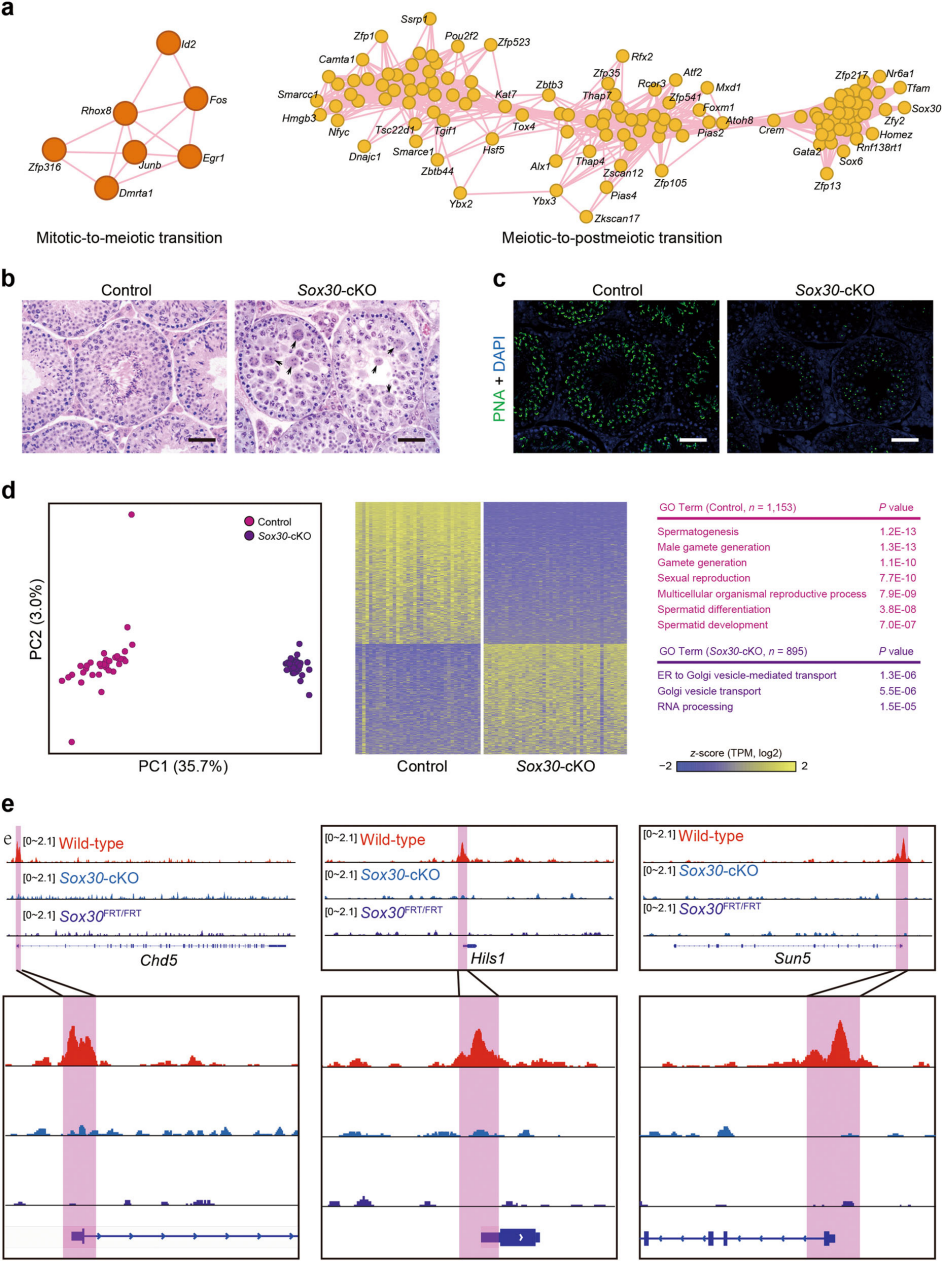
Transcriptional regulation in mouse spermatogenesis. **a** Gene regulation network analysis of mitotic-to-meiotic transition (left panel) and meiotic-to-postmeiotic transition (right panel). Edges indicate interactions between transcriptional factors (TFs). Circles indicate the TFs. Only TFs with high correlation and at least three edges are shown. **b** H&E staining of wild-type control and *Sox30*-cKO testis sections at 8 weeks old. Arrows indicate multinucleated giant cells. Scale bar, 50 μm. **c** Testis sections from adult wild-type control and *Sox30*-cKO mice were immunostained with fluorescence dye-labeled peanut lectin (PNA, green) for acrosomes, and DAPI (blue). Scale bar, 50 μm. **d** Principal component analysis (PCA) (left panel) of steps 3–4 spermatids in *Sox30*-cKO and wild-type control mice. The cells from *Sox30*-cKO mice are shown in purple, whereas cells from wild-type mice are in fuchsia. The variation values of PC1 and PC2 are 35.7% and 3.0%, respectively. Heatmap (middle panel) showing the distinct gene expression characteristics between spermatogenic cells of *Sox30*-cKO and wild-type control mice. Color key from yellow to blue represents the relative gene expression level from high to low. GO analysis (right panel) indicates the potential functions of DEGs in wild-type (fuchsia) and *Sox30*-cKO mice (purple). **e** Snapshots showing peak density by ChIP-seq of the representitive genes, *Cdh5* (left panel), *Hils1* (middle panel) and *Sun5* (right panel) in wild-type, *Sox30*-cKO and *Sox30*^FRT/FRT^ mice, respectively. Zoomed-in peak is shown in the bottom panel

As shown above, spermatogenic cells exhibit stringent developmental stage-specific and cell type-specific transcription of genes required for mitotic, meiotic and postmeiotic processes. Transcription factors (TFs) are thought to play crucial roles to ensure spatiotemporal transcription in spermatogenic cells, thereby orchestrating spermatogenesis. For example, two key TFs, Ume6/Ime1 and Ndt80, control sporulation in yeast.^37^ To identify essential TFs that may regulate the two main transitions, mitotic-to-meiotic transition and meiotic-to-postmeiotic transition, we analyzed the co-expression networks of TFs between clusters C1 and C2 (mitotic-to-meiotic transition), and between clusters C4 and C6 (meiotic-to-postmeiotic transtion) (Fig. 5a; see Materials and Methods). Our analysis predicted that c-*fos*/c-*jun* and *Zfp316* may play roles in the mitotic-to-meiotic transition, whereas many other TFs could potentially regulate spermatid development (Fig. 5a). Of these TFs, *Crem*, and *Rfx2* have been previously shown to be critical for spermiogenesis,^4,38^ whereas several other putative TFs (such as *Sox30* and *Zfp541*) might also function during spermiogenesis. Based on gene expression specificity and ranking, we chose *Sox30* gene for further functional study.

*Sox30*, a member of the *Sox* (SRY-related HMG-box) gene family, is highly expressed in pachytene spermatocytes and round spermatids but not in somatic cells (Supplementary information, Fig. S22a, b). A *Sox30*-knockout (KO) mouse line was generated using a KO-first strategy, which generates a global KO at the RNA processing level and also allows for the generation of conditional KO alleles by combining FLP/FRT and Cre/loxP systems (Supplementary information, Fig. S22c). Importantly, conditional *Sox30* KO alleles lacking exons 2 and 3 produce a mutant Sox30 protein without the DNA-binding domain because exons 2 and 3 of *Sox30* gene encode the HMG box domain. We generated both germ cell-specific *Sox30* KO (by crossing with *Stra8*-*GFPCre* mice; hereafter referred to as *Sox30*-cKO) and a global *Sox30* KO line. RNA-seq confirmed that *Sox30* RNA was absent in the global KO mice, whereas *Sox30*-cKO germ cells still expressed the exons 2/3-deleted *Sox30* RNA (Supplementary information, Fig. S22d). Both *Sox30*-cKO and global *Sox30* KO mice showed male sterility. In the *Sox30*-cKO mouse testes, we observed an absence of elongating and elongated spermatids and many multinucleated giant cells (formed by arrested spermatids), revealing an arrest of early spermatid development (Fig. 5b). This is consistent with a recent study.^39^ Immunostaining further demonstrated that haploid cells underwent a complete arrest before appearance of steps 3–4 round spermatids (Fig. 5c). The global *Sox30* KO mice showed the same defects as the *Sox30*-cKO mice, indicating that deletion of the DNA-binding domain is sufficient to elicit the spermatid phenotypes (Supplementary information, Fig. S22e, f). To determine the consequences of the loss of *Sox30*, we profiled 49 individual round spermatids at step 3 from the *Sox30*-cKO testes. After QC filtering, we retained 48 single-cell transcriptomes for subsequent analysis (Supplementary information, Table S1). Compared with 37 individual round spermatids at steps 3–4, we identified 2,048 (1,153 down-regulated and 895 up-regulated) significant differentially expressed genes upon *Sox30* deficiency (Fig. 5d and Supplementary information, Table S6). Notably, GO analysis revealed that genes required for spermatid development were highly enriched in the down-regulated genes, whereas genes involved in RNA processing and Golgi vesicle transport were specifically enriched in the up-regulated genes (Fig. 5d). We performed chromatin immunoprecipitation followed by high-throughput sequencing (ChIP-seq) to identify genes that are directly controlled by SOX30 in early spermatids, using steps 2 and 3 spermatids. We identified 3,129 significant peaks corresponding to SOX30 binding (see Materials and Methods). Of these peaks, 1,704 (54.5%) peaks were located within potential promoter regions (Supplementary information, Fig. S22g, h), suggesting that they might be SOX30 target genes. Among these potential targets, 233 and 127 were down-regulated and up-regulated in *Sox30*-deficient round spermatids, respectively. GO analysis of down-regulated targets revealed a significant enrichment in genes involved in spermatid differentiation including *Chd5*, *Hils1*, and *Sun5* (Fig. 5e). Taken together, these results demonstrate a crucial role of a novel transcriptional regulator Sox30 in spermatid development. This again highlights the value of our dataset as a rich resource of candidates that regulate spermatogenesis.

### Alternative splicing patterns during mouse spermatogenesis

**Fig. 6:**
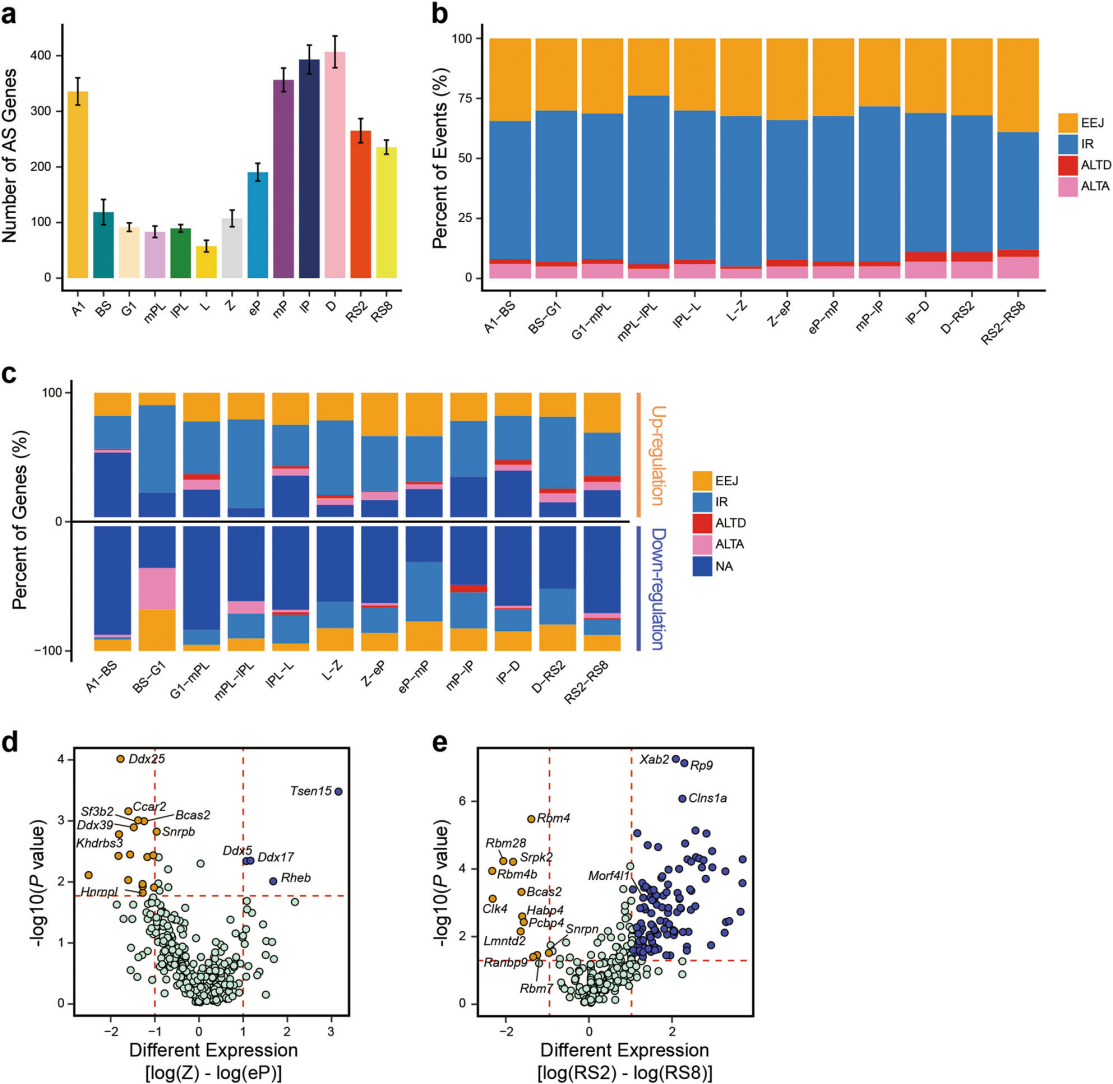
Characterization of dynamic patterns of alternative splicing and its regulation during spermatogenesis. **a** Bar plot showing the average number of genes with at least two alternative splicing (AS) events at different stages. Error bar represents mean ± SEM. **b** Stacked bar plot indicating the ratio of AS event changes when comparing two contiguous stages. EEJ exon-exon junction, IR intron retention, ALTD alternative donors, ALTA alternative acceptors. The AS event is classified by its status in the latter stage. **c** Stacked bar plot showing the ratio of AS status in up- (upper panel) and down-regulated genes that have AS events shared between two consecutive stages. For up-regulated genes, the AS status is shown according to the latter stage; for down-regulated genes, the AS status is shown according to the former stage. NA, no AS status. See Materials and Methods for details. **d–e** Volcano plots showing differentially expressed splicing regulator genes in Z against eP (**d**) and RS2 against RS8 (**e**). The genes in purple (FDR ≤ 0.05 and average difference ≥ 1) or orange (FDR ≤ 0.05 and average difference ≤ –1) are selected splicing regulator genes

In addition to transcriptional regulation, spermatogenic cells are known to have high levels of AS variants.^6,16,17^ We identified 9,701 AS events (including exon-exon junction (EEJ), intron retention (IR), alternative donors (ALTD), and alternative acceptors (ALTA)) in 2,830 genes in spermatogenic cells. In addition, number change patterns of genes within splice variants are strikingly similar to gene expression patterns during spermatogenesis, suggesting that AS is the main contributor of transcriptome complexity in the male germlines (Figs. 2b, 6a and Supplementary information, Table S7; see Materials and Methods). We then determined the distribution of four AS events in each type of spermatogenic cells (Supplementary information, Fig. S23a and Table S7). It is of note that IR and EEJ were the most common AS events detected in the spermatogenic cells we analyzed (Supplementary information, Fig. S23a). We further identified AS changes between all the consecutive stages using merged single-cell data, and found that the most frequent change was in the number of IR events regardless of mitotic, meiotic and postmeiotic cells (see Materials and Methods). This suggests that IR change is a predominant feature of splicing regulation in male germ cell development (Fig. 6b, Supplementary information, Fig. S23b and Table S7). To determine the contribution of changes in splicing patterns (types and levels) to the overall expression levels of genes, we compared the differentially expressed genes (those upregulated and downregulated) with their splicing patterns between the consecutive spermatogenic cells (see Materials and Methods). We found that splicing changes from all to none (i.e., an overall loss of splicing) were associated with downregulation of genes, whereas an increase in IR events was highly enriched in up-regulated genes (Fig. 6c).

In testis, four major *Spo11* isoforms are produced by AS: *Spo11α* (exon 2-skipped, EEJ), *Spo11β*, *Spo11-3* (unknown function, EEJ), and *Spo11-4* (IR). It has been suggested that *Spo11β* introduces DSB at leptonema, whereas *Spo11α* is critical for XY pairing at pachynema.^40^ To determine whether splicing regulation of *Spo11* plays a role in meiosis, we analyzed splicing pattern switches across meiotic cells. Interestingly, we found that the *Spo11β* isoform was mainly generated in mid-preleptotene and leptotene spermatocytes, the IR isoform *Spo11-4* was in late preleptotene, early pachytene, and mid-pachytene spermatocytes, and the exon 2-skipped isoform *Spo11α* was in mid-pachytene spermatocytes (Supplementary information, Table S7). The isoforms *Spo11β* and *Spo11α* were present in the cells where their products function, further highlighting the important role of splicing regulation during meiosis.

Regulation of AS relies on combinatorial interactions between splicing regulators.^17^ We identified 180 splicing regulator genes that were differentially expressed between spermatogenic cells (Supplementary information, Table S7; see Materials and Methods). Of these factors, *Ranbp9* and *Morf4l1* (*Mrg15*), which are differentially expressed between step 2 and step 8 round spermatids, have been shown to be essential for spermiogenesis (Fig. 6d, e), demonstrating the potential value of this dataset.^41,42^ Interestingly, we observed that a splicing factor hnRNPL, whose mutation is associated with non-obstructive azoospermia in humans,^43^ was significantly differentially expressed between zygotene and early pachytene spermatocytes, indicating that it could control meiosis through splicing regulation of target genes.

### Transcription patterns of genes on the sex chromosomes during mouse spermatogenesis

**Fig. 7:**
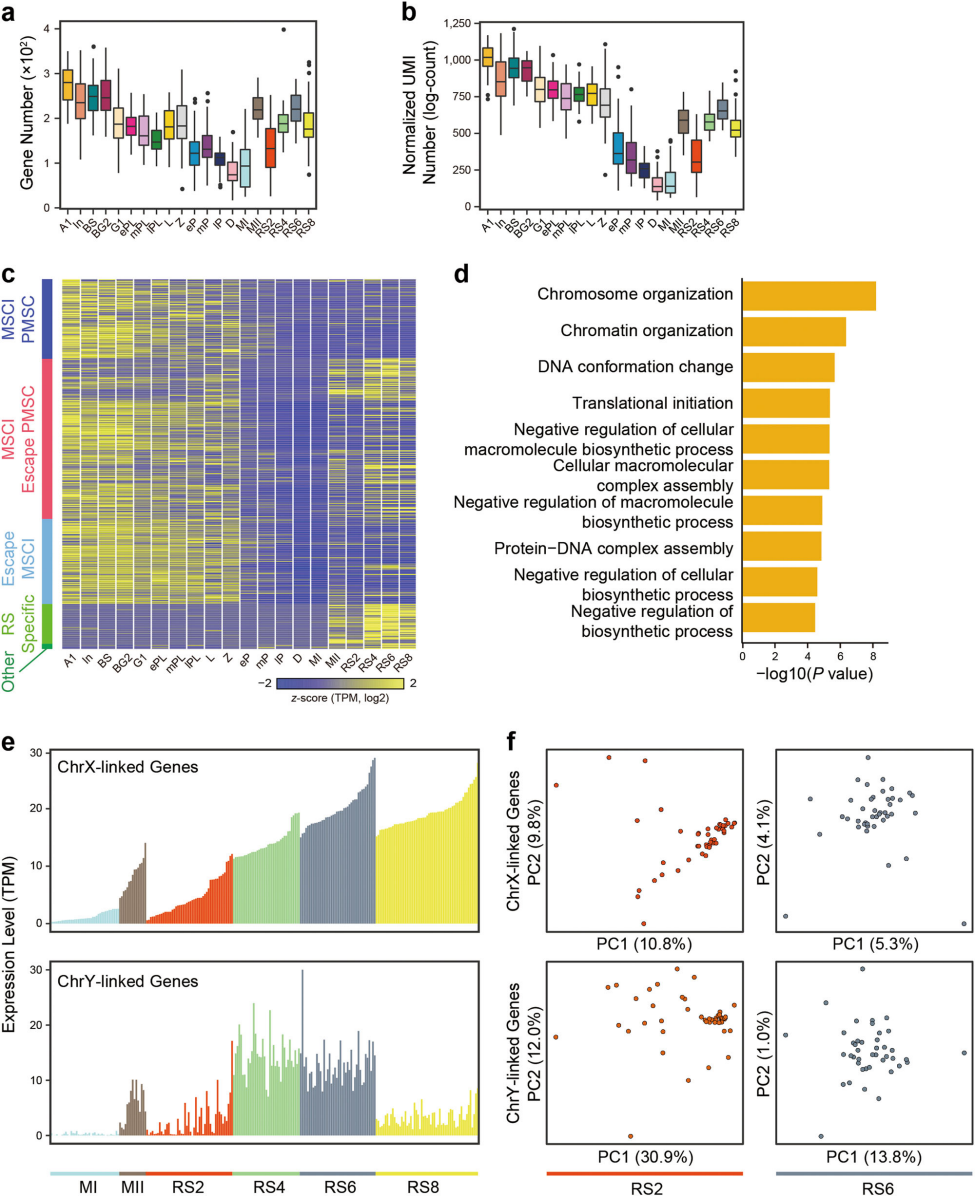
Dynamic expression patterns of sex chromosome-linked genes during spermatogenesis. **a** Boxplots showing the numbers of protein-coding genes on sex chromosomes expressed in each individual cell at 20 developmental stages. **b** Boxplots indicating the normalized UMI counts of protein-coding genes on sex chromosomes expressed in each individual cell at 20 developmental stages. **c** Heatmap showing five groups of sex chromosome-linked gene expression patterns. The colored bars on the left from top to bottom represent MSCI PMSC, MSCI escape PMSC, escape MSCI, RS specific, and other, respectively. The classification standard of five groups above, see Materials and Methods for details. Color key from yellow to blue represents the relative gene expression level from high to low. **d** GO analysis of escaped MSCI genes. **e** Bar plots showing X chromosome-linked (top panel) and Y chromosome-linked (bottom panel) gene expression levels in MI, MII, RS2, RS4, RS6, and RS8 stages. **f** Principal component analysis (PCA) showing the distribution of cells from RS2 and RS6 based on chromosome X (ChrX)-linked genes (top panel) and chromosome Y (ChrY)-linked genes (bottom panel)

In mammals, the patterns of gene expression on sex chromosomes are complicated during spermatogenesis. The transcriptional status of the sex chromosomes at each stage of spermatogenesis remains to be fully investigated. We took advantage of our synchronous single spermatogenic cells that sampled the mitotic, premeiotic, meiotic and postmeiotic stages, to determine the temporal pattern of sex chromosome-linked gene expression during mouse spermatogenesis. Overall, we identified that 637 out of 817 (78.0%) X chromosome protein-coding genes, and 18 out of 40 (45.0%) Y chromosome protein-coding genes were expressed in spermatogenic cells (Supplementary information, Table S8; see Materials and Methods). The expression pattern of these genes throughout spermatogenesis is shown in Fig. 7a, b. We found that an abundance of sex chromosome-linked protein-coding genes were expressed in spermatogonia as previously reported,^44,45^ whereas the lowest numbers and expression levels of sex chromosome-linked protein-coding genes were detected in pachytene and diplotene spermatocytes (Fig. 7a, b; Supplementary information, Fig. S24a and Table S8). It is known that the transcriptional activity of sex chromosomes is silenced during mammalian meiosis prophase I, a process known as MSCI.^46^ These results thus provide evidence of the global effects of robust MSCI on transcription. As a control, there was no relationship between gene expression and MSCI on autosomes (Supplementary information, Fig. S24b). However, we still detected a large number (150) of sex chromosome-linked protein-coding genes in later stage meiotic prophase I cells (from early pachytene to diplotene spermatocytes) (Fig. 7c and Supplementary information, Table S8). Close inspection of these transcript patterns showed that most of them were absent in diplotene but present in pachytene spermatocytes (Supplementary information, Table S8). We thus defined the sex chromosome-linked genes that are expressed in spermatogenic cells at any stage before early pachynema but not expressed in the subsequent diplonema, as MSCI genes. We found that 575 sex chromosome-linked protein-coding genes were expressed in spermatogenic cells at some stage before early pachynema. Of these, 425 genes (73.9%) were subjected to MSCI, whereas 150 (26.1%) genes seemed to escape from MSCI, but may instead represent stable transcripts persisting along spermatocytes, as suggested by previous pachytene cell population-based studies (Fig. 7c, Supplementary information, Fig. S24c and Table S8).^7^ For the MSCI genes, 154 out of 425 (36.2%) genes were absent in both pachytene and diplotene spermatocytes (Supplementary information, Fig. S24d), whereas the remaining 271 genes (63.8%) were still expressed in pachytene cells (Supplementary information, Fig. S24e). We further showed that expression of these 271 genes in pachytene spermatocytes had distinct patterns (Supplementary information, Fig. S24e). Notably, 140 (32.9%) MSCI genes remained silent in postmeiotic cells, indicating that these genes are also subjected to postmeiotic sex chromatin (PMSC) silencing.^47,48^ Nevertheless, we found that the majority (67.1%) of MSCI genes showed varying extents of reactivation in MII spermatocytes and round spermatids (from step 1 to step 8), indicating that these protein-coding transcripts escape from PMSC silencing (Fig. 7c, Supplementary information, Fig. S24d, e and Table S8). Interestingly, essentially all sex chromosome-linked protein-coding genes (148) expressed in diplotene spermatocytes remained expressed in postmeiotic round spermatids, except for *Chic1* and *Nkap* (Fig. 7c and Supplementary information, Table S8). Among these transcripts, many encode proteins required for sperm function, such as *Taf7l*.^49^ Moreover, GO analyses indicated that these “silencing escaped” genes are important for chromatin remodeling (Fig. 7d and Supplementary information, Table S8). In addition to these transcripts, we identified 71 sex chromosome-linked genes that were expressed only in round spermatids (Supplementary information, Table S8).

### Expression pattern of sex chromosome-linked genes demonstrates that postmeiotic spermatids share transcripts

Meiotic recombination and chromosome segregation give rise to genetically different spermatids, including X chromosome- and Y chromosome-bearing haploid spermatids. Several studies have previously suggested that spermatids share specific gene transcripts via connecting intercellular bridges.^50–52^ However, how extensive this sharing of postmeiotic transcripts is remains unclear due to the difficulty in comprehensively examining gene transcripts in single cells. The single-cell RNA-seq data for a large number of postmeiotic cells here provided the opportunity to address this question. As described above, spermatids express a large number of sex chromosome-linked genes. We thus used sex chromosome-linked transcripts for the analysis because only half of the spermatids harbor X chromosome, and the other half receive Y chromosome.

We first tested whether X chromosome-bearing spermatids carried more X chromosome-linked transcripts than Y chromosome-bearing spermatids, and vice versa. Interestingly, we found that all individual spermatids from the same stage exhibited similar numbers or levels of sex chromosome-linked transcripts, indicating that the transcripts of sex chromosome-linked genes are equally distributed between spermatids in the same stage (Fig. 7e). In addition, we observed that metaphase II spermatocytes also showed highly similar sex chromosome-linked gene transcripts between X chromosome-bearing and Y chromosome-bearing spermatocytes (Fig. 7e). We next performed PCA of sex chromosome-linked gene expression profiles on spermatids at all developmental stages to determine whether X chromosome-bearing spermatids could be distinguished from Y chromosome-bearing spermatids. As shown in Fig. 7f, the spermatids from the same stage could not be separated by PCA analysis, further demonstrating that the spermatids share transcripts. Altogether, our data provide direct and quantitative evidence that global sharing of transcripts can occur due to intercellular bridges connecting spermatids, resulting in phenotypic equivalence of genetically distinct spermatids.

## Discussion

Despite the clear biological importance for male germ cell development, current knowledge on the underlying molecular mechanisms of the cellular fate transition and stage-specific gene regulation during mammalian spermatogenesis remains largely incomplete. This is partly due to technical limitations of analyzing spermatogenic cell populations at heterogeneous and mixed substages. In the current study, we were able to overcome many of these limitations. We established an approach to successfully purify all types of homogeneous male germ cells from mice at both single-cell resolution and population levels. The ability to isolate homogeneous spermatogenic cells allowed us to not only accurately decipher the involvement of molecules in cellular state transitions and stage-specific gene regulation, but also to utilize these cells for functional characterization. With single-cell RNA-seq, we generated a high-resolution atlas of the transcriptomes with comprehensive coverage of male germ cells during mouse spermatogenesis. This level of resolution enabled us to define seven distinct main clusters within the twenty stages of spermatogenic cells. Notably, we further revealed that the transcriptional switch driving the mitotic-to-meiotic transition might occur before early preleptotene. Here, a key unresolved issue is the regulator(s) that control this transition; our study suggests c-*fos*/c-*jun* and *Zfp316* as potential candidates. Unexpectedly, round spermatids at different stages show remarkably different gene expression profiles. Moreover, our study revealed the detailed changes in splicing and the differential expression of splicing regulators that occur across male germ cell development. Our dataset uncovered extensive and previously uncharacterized dynamic processes in AS during spermatogenesis, further underscoring the importance of posttranscriptional regulation for mammalian spermatogenesis. Earlier studies have observed AS changes around meiosis.^6,16,17^ However, these studies did not address global splicing and splicing regulator changes during spermatogenesis, especially at the mitotic-to-meiotic transition. In addition, our findings indicated that, despite low expression levels at the single-cell resolution, some sex chromosome-linked genes escape from MSCI. These genes could be crucial for chromatin remodeling during spermatid differentiation. Furthermore, our transcriptomic analyses have guided us to define novel key regulators in spermatogenesis. For example, we demonstrated that *Fbxo47* functions in meiotic recombination, and that *Sox30* drives spermiogenesis through its direct transcriptional targets. We also directly demonstrated that postmeiotic spermatids share global transcripts. Thus, this study showcases the power of exploring these data and paves the way towards deepening our understanding of the regulatory basis of male germ cell development, highlighting the importance of oversampling and purification of homogeneous cell populations for defining the dynamic processes and regulatory programs of spermatogenesis.

Our single-cell dataset also permitted us to screen and validate a set of surface markers for purifying round spermatids at different steps, and to deduce the different developmental potentials of embryos generated by these round spermatids. It will be of interest to determine whether those markers could be used to isolate round spermatids at different stages from human. In this study, we also discovered that early stage round spermatids have much lower embryo developmental potential compared to late stage round spermatids. Thus, the differentiation of round spermatids could be key to their function as male gametes, challenging the approach to use a mixture of round spermatids for ROSI. Our results provide evidence that maturation of round spermatids impacts on embryo developmental potential, and this is relevant for considerations not only on low success rates of clinical ROSI^35,36^ but also on human embryo quality. Future clinical and basic animal research should evaluate whether there is a relationship between embryo quality and ROSI using early or later stage round spermatids.

In conclusion, we have derived precise and previously uncharacterized, specific molecular signatures for each subtype or cluster of spermatogenic cells during spermatogenesis, providing a rich resource for further study in germ cell biology. Combined with other massively parallel sequencing techniques (such as ChIP-seq, ATAC-seq, Hi-C, and ribosome profiling), our approach can be readily applied to further dissection of the complex molecular regulation networks, including dynamic epigenetic signatures, chromatin remodeling dynamics, posttranscriptional regulation, and translational control, during mouse spermatogenesis.

## Materials and methods

### Mice

Mice used in this study were as follows: *Lin28*-YFP, *Vasa*-dTomato, *Stra8*-GFPCre, *Sox30*^tm1a(KOMP)Wtsi^, and *Fbxo47*^tm1a(EUCOMM)Wtsi^. All mice described were maintained on the C57BL/6 J (B6) background. The *Stra8*-GFPCre mouse line was described previously.^32^ The *Lin28*-YFP and *Vasa*-dTomato mouse lines were generated by the CRISPR/Cas9 technology. For the *Lin28*-YFP line, a cDNA encoding the YFP was inserted into the last coding exon of *Lin28a*, and a 2 A peptide sequence was included to link *Lin28a* and YFP to allow expression of both genes. For the *Vasa*-dTomato line, a cDNA encoding dTomato was inserted into the last exon of *Vasa*, and a 2 A peptide sequence was included to link *Vasa* and dTomato. The *Lin28*-YFP and *Vasa*-dTomato lines were generated by Shanghai Biomodel Organism Co., Ltd. *Sox30*^tm1a(KOMP)Wtsi^ mouse sperm was obtained from Baylor College of Medicine. *Fbxo47*^tm1a(EUCOMM)Wtsi^ mice were obtained from University of Veterinary Medicine Vienna. All animal experiments were conducted in accordance with the guidelines in the Animal Care and Use Committee at Shanghai Institute of Biochemistry and Cell Biology, Chinese Academy of Science. Genotyping of *Lin28*-YFP or *Vasa*-dTomato mice was done by PCR on tail genomic DNA. Primers for PCR genotyping of *Lin28*-YFP mice are: Lin28_YFP_F: CCC CAG TTC TCA GGG AAA GCC; Lin28_YFP_wtR: CCA CCC TTA CCC CCA CTT TCT; Lin28_YFP_kiR: TGA ACT TGT GGC CGT TTA CGT. Primers for PCR genotyping of Vasa-dTomato mice were: Vasa_dTomato_F: AGA CTT CAC AGG ATT ACA TTG; Vasa_dTomato_wtR: AAA GCA CAT CAC ATC CTA TTG; Vasa_dTomato_kiR: CCC TTG CTC ACC ATA GGA CCA.

### Synchronous spermatogenesis

*Lin28*-YFP mice were crossed with *Vasa*-dTomato mice to generate the mice carrying both *Lin28*-YFP and *Vasa*-dTomato alleles for synchronous spermatogenesis. *Stra8*-GFPCre mice were used for synchronous spermatogenesis to generate type A1 spermatogonia. Spermatogenesis was synchronized as previously described with modifications.^21^ Briefly, 2-dpp mice were pipette fed 100 μg/g body weight WIN 18,446, suspended in 1% gum tragacanth, for 7 consecutive days.^21^ On day 8 of WIN 18,446 treatments, these animals received an i.p. injection of RA (33 μg/g body weight) in dimethyl sulfoxide (DMSO), and were then left to recover for sample collections at the given 20 time points (Supplementary information, Table S1). The testicular tissues at the 20 time points were collected and determined for synchronous efficiency using electron microscopy, histological analyses, chromosome spreading, and immunohistochemical analyses. For cell cycle analysis, animals also received an i.p. injection of 5-ethynyl-2′-deoxyuridine (EdU; 50 μg/g body weight in PBS) 2 h prior to euthanization. EdU incorporation was detected using the Click-It EdU Alexa Fluor 594 Imaging Kit according to the manufacturer’s protocol as previously described.^53^

### Transmission electron microscopy

Fresh testes were fixed in 2.5% (Vol/Vol) glutaraldehyde in 0.1 M phosphate buffer (PB), pH 7.4, for 2 h at 4 °C, washed with PB, postfixed in 2% OsO4 for 1.5 h, dehydrated in a graded ethanol series before being transferred to acetone, and embedded in Poly/Bed 812. Ultrathin sections were taken with a Leica EM UC7 ultramicrotome (Leica, Inc.), doubly stained with uranyl acetate and Reynold’s lead citrate, and then imaged on a FEI Tecnai G2 Spirit TEM (FEI Company) at 120-kV accelerating voltage.

### Surface spreading and immunofluorescence

Chromosome spreading and immunofluorescence were performed as described.^54–56^ The spreading nuclei were immunostained with rabbit anti-SYCP3 (1:500) and mouse anti-γH2AX (1:500) antibodies, detected with Alexa Fluor 488-conjugated or 594-conjugated secondary antibodies (1:500), mounted in Prolong Gold Antifade medium with DAPI, and then analyzed by a fluorescence microscope. According to SYCP3 labeled axial/lateral element characteristics, γH2AX staining and DAPI-stained heterochromatin pattern, spermatocytes in spreading were classified as preleptotene, leptotene, zygotene, and pachytene according to standard protocols.^54^ More than 200 cells were counted per time point unless otherwise noted.

### Histological and immunohistochemical analysis

Testes were fixed in Bouin’s buffer or 4% paraformaldehyde (PFA), embedded in paraffin and sectioned. Sections were deparaffinized, rehydrated, and stained with Hematoxylin and Eosin (H&E). For immunofluorescence analysis, the following primary antibodies were used: goat anti-GFP (1:500; Abcam), rabbit anti-RFP (1:200; Rockland), rabbit anti-Lin28a (1:100; Abcam), rabbit anti-STRA8 (1:200; gift from Michael Griswold), mouse anti-γH2AX (1:500; Millipore), guinea pig anti-H1t (1:500; gift from Dr. M. Handel at Jackson Laboratory), rabbit anti-SOX30 (1:200; Abclonal), rabbit anti-VASA (1:200; Abcam), rabbit anti-DMC1 (1:100; Santa Cruz Biotechnology, Inc.), rabbit anti-pH3 (1:500; Millipore), FITC-conjugated peanut agglutinin (1:500; Sigma) and peanut agglutinin (1:500; VECTOR).

### Isolation of spermatogenic cells

We isolated twenty different subtypes of spermatogenic cells from spermatogenesis synchronous mice with *Lin28*-YFP and *Vasa*-dTomato alleles as described above. After synchronization, testes from mice at the given time point were collected in PBS and placed on ice. After removal of the tunica albuginea, the testes were incubated in 5 mL of PBS containing 120 U/mL of collagenase type I at 32 °C with gentle agitation for 5 min. The dispersed seminiferous tubules were further digested with 5 mL of 0.25% trypsin, 0.1 mL of DNase I (5 mg/mL) by pipetting gently several times at 32 °C for 8 min, and then terminated by adding 0.5 mL of fetal bovine serum (FBS) to inactivate trypsin. Following two-step enzymatic digestion, the dissociated testicular cell suspension was then filtered through a PBS-prewetted cellular filter with pore size of 70 μm. The cell suspension was centrifuged at 500× *g* for 5 min at 4 °C, and the supernatant was carefully removed from the pellet. The cells in the pellet were resuspended at a concentration of 1 × 10^6^ cells/mL in DMEM with Hoechst 33342 (3 mg/mL) and 5 μl DNase I. For collection of a single cell, the cell suspension was placed onto a 6-well plate at low density for 20 min at room temperature followed by picking single cells based on their fluorescent label using Unipick system. For FACS, the cell suspension was rotated for 20 min at 32 °C in the oven at 10 r.p.m./min speed, then centrifuged at 500× *g* for 5 min at 4 °C, and resuspended in 0.3–1 mL DMEM for sorting. Cell populations were collected based on their fluorescent label with Hoechst 33342 staining using FACS.

### Isolation and identification of round spermatids

The testicular cell suspension was collected from adult wild-type mice and stained by Hoechst with a similar protocol as described above. The cell suspension was centrifuged and the pellet was resuspended at a concentration of 1 × 10^6^ cells/40 μL, followed by incubating with APC-conjugated anti-CD63 antibody (0.1 μg/10^6^ cells) for 30 min on ice and washed twice with DMEM. CD63^–^ and CD63^high^ round spermatids (1 N) were isolated according to Hoechst 33342 staining and APC fluorescent label using FACS. Cells were collected and further examined by PNA staining as described above.

### Whole-mount immunohistochemistry

Mouse testes were removed from the tunica albuginea, and untangled seminiferous tubules were fixed in 4% PFA with 0.5 mM CaCl_2_, and PBS on ice for 4 h. The seminiferous tubules were washed in PBS with 0.2% NP40 for 20 min, and dehydrated through a graded methanol series (25%, 50%, 75%, and 100%) in PBS containing 0.1% Tween 20 (PBST) on ice for 5 min each. After rehydration in PBST for 5 min twice, the tubules were blocked in blocking buffer (1% BSA and 4% donkey serum in PBST) for 1 h, and incubated with primary antibodies against GFP (1:500), LIN28A (1:100) in blocking buffer at 4 °C overnight. After washing in PBST, the tubles were incubated with Alexa Fluor 594-conjugated donkey secondary antibody for 2 h at room temperature. The tubules were then washed in PBST, mounted, and observed using confocal microscopy.

### Intracytoplasmic round spermatid injection

ROSI was performed as described previously.^57^ In brief, matured MII oocytes were collected from superovulated B6D2F1 females (6–8 weeks). Oocytes were pre-activated by calcium-free CZB medium containing 10 mM SrCl_2_ for 30 min before microinjection. The sorted round spermatids were then injected into the oocytes with a Piezo-driven pipette. All reconstructed embryos were activated using SrCl_2_ in a similar manner described above for 3 h. Completely activated embryos were cultured in KSOM medium with amino acids at 37 °C under 5% CO_2_ in air for 3.5 days to reach blastocyst stage.

### Chromatin immunoprecipitation (ChIP) and ChIP-seq

The sorted synchronous round spermatids from wild-type, *Sox30*-cKO and *Sox30*^FRT/FRT^ mice were crosslinked with 1% formaldehyde for 10 min at room temperature, and quenched by adding glycine to a final concentration of 0.25 M for 5 min. Cells were collected and washed twice with cold PBS containing 1× protease inhibitor cocktail. Cell pellets were lysed in lysis buffer (0.1% SDS, 20 mM Tris-HCl, pH 8.0, 500 mM NaCl, 1 mM EDTA, 1% Triton X-100 and 1× protease inhibitor cocktail) for 10 min on ice, and sonicated with Qsonica to obtain a chromatin size of 200–500 bp. SOX30 antibody (5 μg) was incubated with chromatin samples overnight at 4 °C. Spike-in antibody (0.5 μg) and spike-in chromatin (5 ng) were used in this ChIP assay according to the manufacturer’s guidelines. Then the protein-DNA complexes were immobilized to 30 μL protein A/G beads, followed by washing twice with lysis buffer, 3 times with low salt buffer (10 mM Tris-HCl, 250 mM LiCl, 1 mM EDTA, 0.5% NP40, 0.5% Na-deoxylcholate and 1× protease inhibitor cocktail) and once with 10 mM Tris-HCl, pH 8.0. Decrosslinking was carried out in elution buffer (50 mM Tris-HCl, pH 8.0, 10 mM EDTA, 1% SDS and 20 μg Proteinase K) at 65 °C for 3 h. DNA samples were extracted with Phenol-Chloroform, analyzed using real-time PCR and prepared for deep sequencing according to the manufacturer’s guidelines. Libraries were sequenced using paired-end reads on an Illumina HiSeq X10.

### Single-cell RNA-seq

The single-cell RNA-seq was performed based on Smart-seq2 with some modification.^58–60^ The single spermatogenic cells were picked into lysis buffer containing reverse transcription primer. The oligo dT reverse transcription primer was composed of 8 nt sample-specific barcode, 8 nt unique molecular identifiers (UMIs) and 25 nt oligo dT primer (TCAGACGTGTGCTCTTCCGATCT-XXXXXXXX-NNNNNNNN-T25, X standing for barcode and N representing UMI). The cDNAs were synthesized by template switch oligo (TSO) primer and reverse transcription primer, followed by 20 cycles of PCR with 3′P2 primer and IS primer. Samples with different barcodes were pooled together and then purified using Agencount AMPure XP beads. Index sequences were induced by 4 cycles of PCR using biotin-modified index primer and IS primer. After being sheered by covaris S2, the cDNAs were enriched by incubating with Dynabeads MyOneTM Streptavidin C1 beads. The libraries were constructed using KAPA Hyper Prep Kit. After adaptor ligation, the samples were amplified using QP2 and short universal primer.

Full-length RNA-seq libraries were used for alternative splicing analysis. After cDNA synthesis and amplification, the samples were purified and then fragmented individually. We used KAPA Hyper Prep Kit to construct the libraries and Multiplex Oligos for Illumina to amplify the adaptor-ligated product. We sequenced the libraries on Illumina Hiseq 4000 platform in 150 bp pair-end model.

### Quantification and statistical analyses

#### Processing RNA-seq data

Single-cell RNA-seq raw reads were processed to remove TSO sequence, polyA tail sequence, adaptor contaminants and low-quality bases. The clean reads were aligned to mouse genome (mm10, UCSC version) using TopHat (version 2.0.12) with default parameters,^61^ and uniquely mapped reads were counted with HTSeq.^62^ Finally, for a given cell, the number of UMIs represents transcript number of each gene. We quantified gene expression levels with TPM.

To retain high-quality cell, we performed quality control following three standards: mapping rate was > 40%, number of genes detected was > 2000, and UMI number was between 20,000 and 1,000,000 (Supplementary information, Fig. S20a). Once the corresponding transcript number is > 1, we consider that this gene is detected in the individual cell.

Since the variation of sequencing depth and coverage for different single cells potentially exists, we normalized UMI counts with ERCC spike-in by the R package *simpleSingleCell*. Briefly, UMI counts of endogenous genes were normalized with the *computeSumFactors* function (set the parameter *min.mean* to 0.1), and counts of ERCC spike-in were perfomed with the *computeSpikeFactors* function. And then, log-transformed UMI counts were calculated with appropriate size factors.

For full-length single-cell RNA-seq data, clean reads were aligned to mouse genome (mm10, UCSC version) with TopHat as well. We quantified gene expression levels with fragment per kilobase of transcript per million fragments mapped (FPKM) with Cufflinks.^63^ Processing of bulk RNA-seq data was the same as full-length single-cell RNA-seq data.

#### Identification of development clusters

For identification of development relationship of single cells, unsupervised clustering analysis was done. Single-cell RNA-seq expression data was transferred into log_2_(TPM/10 + 1). We performed PCA with 746 variable genes selected by R package Seurat,^64^ and eleven statistically significant PC dimensions (PC1~PC11) were selected for *t*-SNE analysis. Finally, we set the parameter *resolution* to 0.25 for function *FindClusters* in Seurat to identify development clusters. Three-dimension plot of PCA was visualized with R package scatterplot3d.^65^

#### Identification of differentially expressed genes

Dynamic changes of differentially expressed genes (DEGs) between consecutive stages, different stages, or consecutive clusters were identified with function *FindMarkers* in R package Seurat. Single-cell RNA-seq expression data was transferred into log_2_(TPM/10 + 1). DEGs between different cell types were analyzed with function *FindAllMarkers*.

A *t*-test was used to calculate *P* values and adjusted *P* values (false discovery rate, FDR) were calculated with the Benjamini and Hochberg (BH) method. DEGs were defined only if *P* values and FDR were both < 0.05, and a fold change (log_2_-transferred) was > 1. GO analysis was performed with ToppGene using default parameters.^66^

#### Annotation of protein-coding genes and lncRNAs

20,088 protein-coding genes and 11,962 lncRNAs were annotated by GENCODE (mm10, vM15 version).^67^

#### Spermatogenesis-associated GO term gene collection

All spermatogenesis-associated GO term (corresponding to Fig. 6d, e, and Supplementary information, Fig. S21d-l) gene sets were collected from MGI.^68^

#### Surface marker gene collection

Surface marker genes were collected from BD biosciences database (http://www.bdbiosciences.com/documents/cd_marker_handbook.pdf).

#### Co-expression networks of transcriptional factors

We performed co-expression network analysis of transcriptional factors (TFs) with R package igraph.^69^ 1,395 TFs of mouse were selected from AnimalTFDB 2.0^70^ for downstream analysis. Single-cell RNA-seq expression data was transferred into log_2_(TPM/10 + 1). We only considered TFs with the high correlation (for mitotic-to-meiotic transition, correlation was > 0.35; for meiotic-to-postmeiotic transition, correlation was > 0.45) and with at least 3 edges.

#### Processing ChIP-seq data

ChIP-seq raw reads were trimmed to remove adaptor contaminants and low-quality bases using Trimmomatic (version 0.36) with defined parameters (-LEADING:3, -MINKEN:36),^71^ and then reads were aligned to the mouse genome (mm10, UCSC version) using bowtie2 (version 2.2.3) with default parameters.^72^ To obtain uniquely mapped reads, duplicated reads were removed using command filterdup in MACS2 (version 2.1.1) software.^73^ We used command predictd in MACS2 to predict fragment size in all ChIP-seq samples and predicted peaks using command callpeak in MACS2 with defined parameters (-extsize 226, -q 0.001). After filtering, we obtained 3,129 clean peaks in wild-type data.

We visualized ChIP-seq peaks with IGV software^74^ and annotated peaks with *ChIPseeker* R package.^75^

#### Processing alternative splicing data

We selected 5 or 6 high-quality single cells at each development stage for alternative splicing analysis, and obtain 66 single cells from 13 stages. These 66 single cells were sequenced from full-length RNA-seq library, and raw reads were processed with VAST-TOOLS (version 1.1.0).^76^ Reads were aligned to the mouse mm9 genome and genome coordinates were transferred into mm10 genome in VAST-TOOLS.

To detect dynamic changes of AS events between consecutive stages, we merged RNA-seq raw data of single cells from the same stage. Therefore, 66 single cells were merged into 13 samples, and these samples were processed with VAST-TOOLS again.

We detected and quantified four AS types, EEJ, IR, ALTD/Alt5, and ALTA/Alt3, as described before.^77^

#### Identification of differentially expressed splicing regulator genes

When we identified differentially expressed splicing regulator genes, we combined three gene sets collected from MGI (‘regulation of RNA splicing’, ‘RNA splicing’ and ‘spliceosomal complex’) and two additional genes (*Ranbp9* and *Morf4l1*), and thus, 359 genes were used in the downstream analysis.

Sixty-six full-length RNA-seq data were used to identify splicing regulator genes. When we calculated the average expression difference (corresponding to Fig. 6d, e), we calculated (log(mean(exp(*x*)−1)+1)) – (log(mean(exp(*y*)−1)+1)) as R package Seurat where *x* and *y* represented gene expression levels in two groups, respectively. Genes were selected only if *P* values and FDR were both < 0.05, and a fold-change (log_2_-transferred) was > 1.

#### Definition of alternative splicing events and responding genes

For four types of alternative splicing events in 66 single cells or 13 merge samples, we determined the consideration of events with the same standards (modified based on a previous study^77^): 10 ≤ PSI/PSU/PIR ≤ 90 in at least 10% of the cells/samples with sufficient read coverage (if 50 out of 66 cells have sufficient read coverage, the responding threshold is 5 cells), or, 10 ≤ PSI/PSU/PIR ≤ 90 in at least 3 cells/samples with sufficient read coverage. The definition of sufficient read coverage was the same as described before.^77^

We only considered the alternative splicing genes that were subjected to at least 2 types of junctions (EEJ, IR, ALTD, or ALTA) in one given cell/sample.

#### Dynamic changes of alternative splicing events between consecutive stages

We considered dynamic changes of alternative splicing events with merged data (13 samples), and all splicing events were separated into five situations:

1. From stage *x* to stage *y*, splicing events kept still;
2. From stage *x* to stage *y*, splicing events changed but splicing types kept still (e.g., EEJ to EEJ, but splicing situation changes);
3. From stage *x* to stage *y*, splicing types changed (e.g., EEJ to IR);
4. From stage *x* to stage *y*, there was some splicing event in stage *x* but there was not any splicing event in stage *y* (e.g., EEJ to *NA*);
5. From stage *x* to stage *y*, there was not any splicing event in stage *x* but there was some splicing event in stage *y* (e.g., *NA* to EEJ).

We defined genes that met situation (2), (3), (4), and (5) as splicing-regulated genes.

For consideration of dynamic changes of splicing-regulated genes between two consecutive stages, we only considered the splicing event in next stage (if two consecutive stages were from stage *x* to stage *y*, ‘next stage’ referred to as ‘stage *y*’) (Fig. 6b).

When we detected the relationship between expression-regulated genes (up-regulated genes and down-regulated genes between two consecutive stages) and splicing-regulated genes, we selected expression-regulated genes with all cells in corresponding stages, the same as described above. For up-regulated genes, we only considered the splicing event in the next stage; for down-regulated genes, we only considered the splicing event in the previous stage (Fig. 6c).

#### Definition of MSCI-related genes

For a given cell, we considered a expressed gene only if TPM ≥ 1, and based on that, we obtained 18,037 protein-coding genes in all chromosomes (17,269 genes on autochromosomes and 768 on sex chromosomes), and only genes on sex chromosomes were considered for the MSCI-related genes analysis.

To decrease false positive rate of MSCI-related genes, we selected genes from 768 protein-coding genes on sex chromosomes based on the following standard at each development stage: at one given stage, for one given gene, the average TPM was > 1 and TPM ≥ 1 in at least 3 cells at this stage. This was also our definition of ‘expressed gene’ in the MSCI-related gene analysis. We obtained 655 genes in which 637 genes were on X chromosome and 18 genes on Y chromosome.

We defined a ***MSCI Gene*** by the following:

- For a given gene, it was expressed in at least one stage before eP stage (not including eP); i.e.,, the average TPM was > 1 and TPM ≥ 1 in at least 3 cells at this stage;
- This gene was not expressed at stage D; i.e., the average TPM was < 1 or the cell number of TPM ≥ 1 at this stage was < 3.
   If one gene was not expressed among eP, mP and lP stages, we defined that gene as a ***MSCI TypeI*** Gene. Otherwise, we defined that gene as a ***MSCI TypeII*** Gene.
  We also defined ***MSCI Gene*** subclusters by the following: if this gene was expressed in at least one stage after MII stage (including MII), we defined this gene as a ***MSCI PMSC Gene***; otherwise as a ***MSCI Escape PMSC Gene***.
  We defined an ***Escape MSCI Gene*** by the following:
- For a given gene, it was expressed in at least one stage before eP stage (not including eP);
- This gene was expressed at stage D.
  We defined an ***Escape MSCI Gene*** by the following:
- For a given gene, it was expressed in at least one stage before eP stage (not including eP);
- This gene was expressed at stage D.
  We defined a ***RS Specific Gene*** by the following:
- For a given gene, it was not expressed at any stages before MII stage (not including MII);
- This gene was expressed in at least one stage after MII stage (including MII).

For MSCI analysis in lncRNA, we followed the same standards as above.

#### Statistics

All statistics analysis were performed in R language. *P* values were calculated with two-tailed Student’s *t*-test and we considered results with *P* values < 0.05 as significant ones.

### Data and software availability

All 3′ single-cell RNA-seq data, full-length single-cell RNA-seq data and ChIP-seq data are available at the NCBI’s Gene Expression Omnibus (GEO) (http://www.ncbi.nlm.nih.gov/gen/) data repository with the accession ID: GSE107644.

## Electronic supplementary material

Supplementary information, Figure S1

Supplementary information, Figure S2

Supplementary information, Figure S3

Supplementary information, Figure S4

Supplementary information, Figure S5

Supplementary information, Figure S6

Supplementary information, Figure S7

Supplementary information, Figure S8

Supplementary information, Figure S9

Supplementary information, Figure S10

Supplementary information, Figure S11

Supplementary information, Figure S12

Supplementary information, Figure S13

Supplementary information, Figure S14

Supplementary information, Figure S15

Supplementary information, Figure S16

Supplementary information, Figure S17

Supplementary information, Figure S18

Supplementary information, Figure S19

Supplementary information, Figure S20

Supplementary information, Figure S21

Supplementary information, Figure S22

Supplementary information, Figure S23

Supplementary information, Figure S24

Supplementary information, Table S1

Supplementary information, Table S2

Supplementary information, Table S3

Supplementary information, Table S4

Supplementary information, Table S5

Supplementary information, Table S6

Supplementary information, Table S7

Supplementary information, Table S8
