# Supplementary material for: Single-cell RNA-seq uncovers dynamic processes and critical regulators in mouse spermatogenesis
Source: Cell Res. 2018 Jul 30;28(9):879–96. doi: 10.1038/s41422-018-0074-y (PMC6123400; doi:10.1038/s41422-018-0074-y)
Supplement: Supplementary file 1 — Supplementary information, Figure S1 [file 41422_2018_74_MOESM1_ESM.pdf]

Supplementary information, Figure S1

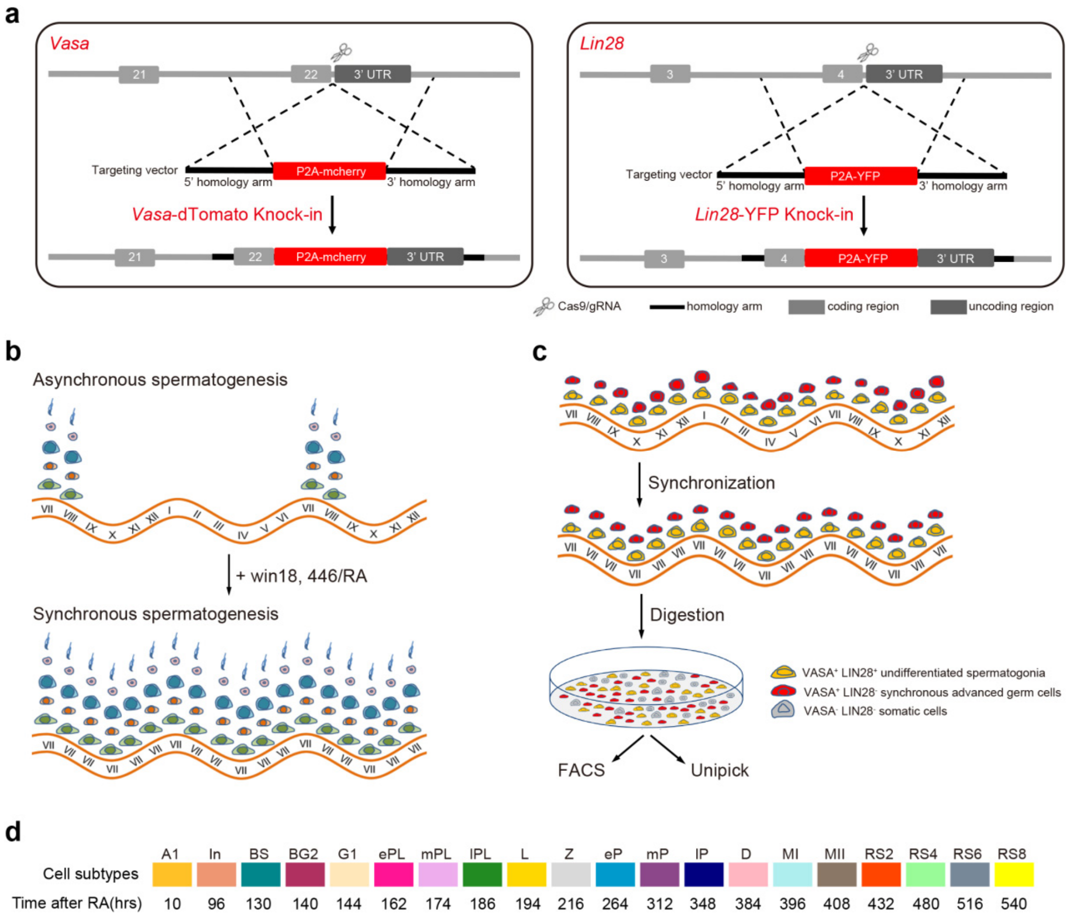

Figure S1 Isolation of specific stage of spermatogenic cells. a Schematic diagram for the *Vasa*-dTomato (left panel) and *Lin28*-YFP (right panel) knockin allele. **b.** After treatment with WIN 18,446/RA, asynchronous spermatogenesis (upper panel, only stage VII and VIII shown) could become synchronous (down panel). After synchronization, no spermatogenic wave is generated and all seminiferous tubules contain the same cellular association. **c** Flowchart for purifying specific stages of spermatogenic cells at both population levels and single-cell resolution. **d** The 20 time-points were given to collect specific stages of spermatogenic cells after RA treatment.
