# Supplementary material for: Single-cell RNA-seq uncovers dynamic processes and critical regulators in mouse spermatogenesis
Source: Cell Res. 2018 Jul 30;28(9):879–96. doi: 10.1038/s41422-018-0074-y (PMC6123400; doi:10.1038/s41422-018-0074-y)
Supplement: Supplementary file 2 — Supplementary information, Figure S2 [file 41422_2018_74_MOESM2_ESM.pdf]

Supplementary information, Figure S2

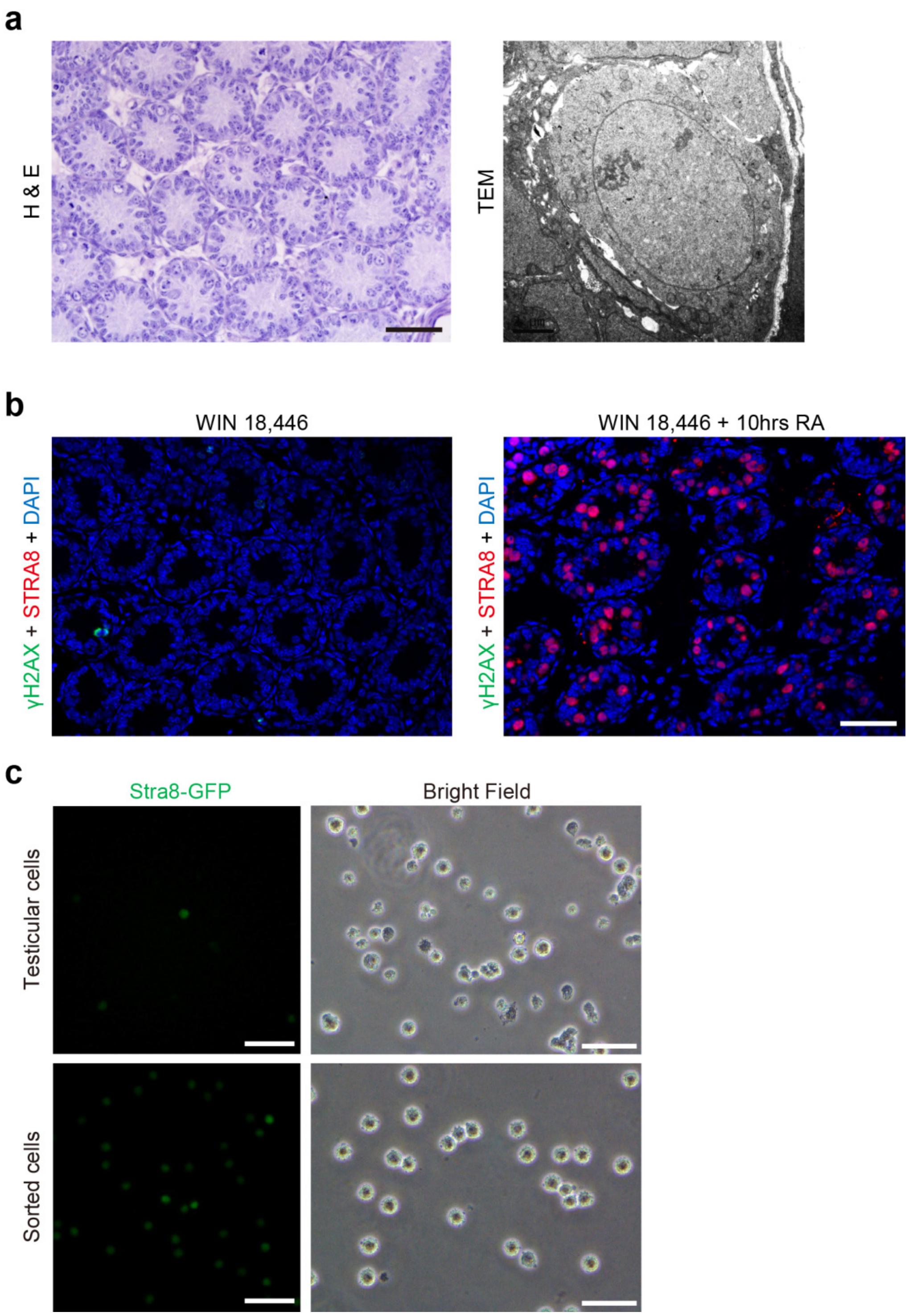

Figure S2 Characterization of A1 spermatogonia (A1) in synchronous spermatogenesis. a Hematoxylin and eosin (H&E) staining and TEM images depict representative cross sections of testes from mice treated with WIN 18,446 followed by an RA injection and allowed to recover for 10 h. **b** Immunohistochemical staining for γH2AX (green), A1 spermatogonia and preleptotene spermatocyte marker STRA8 (red) and DAPI (blue) in sections from mice treated with WIN 18,446-only and WIN 18,446/10 h RA. **c** Representative fluorescence images (observed by fluorescence microscope) and bright field images (observed by inverted phase contrast microscope) of total testicular cell population (upper panel) and sorted cell population by FACS (lower panel). Scale bar, 50 μm. The purity of A1 spermatogonia (A1) is 98.7%.
