# Supplementary material for: Single-cell RNA-seq uncovers dynamic processes and critical regulators in mouse spermatogenesis
Source: Cell Res. 2018 Jul 30;28(9):879–96. doi: 10.1038/s41422-018-0074-y (PMC6123400; doi:10.1038/s41422-018-0074-y)
Supplement: Supplementary file 4 — Supplementary information, Figure S4 [file 41422_2018_74_MOESM4_ESM.pdf]

Supplementary information, Figure S4

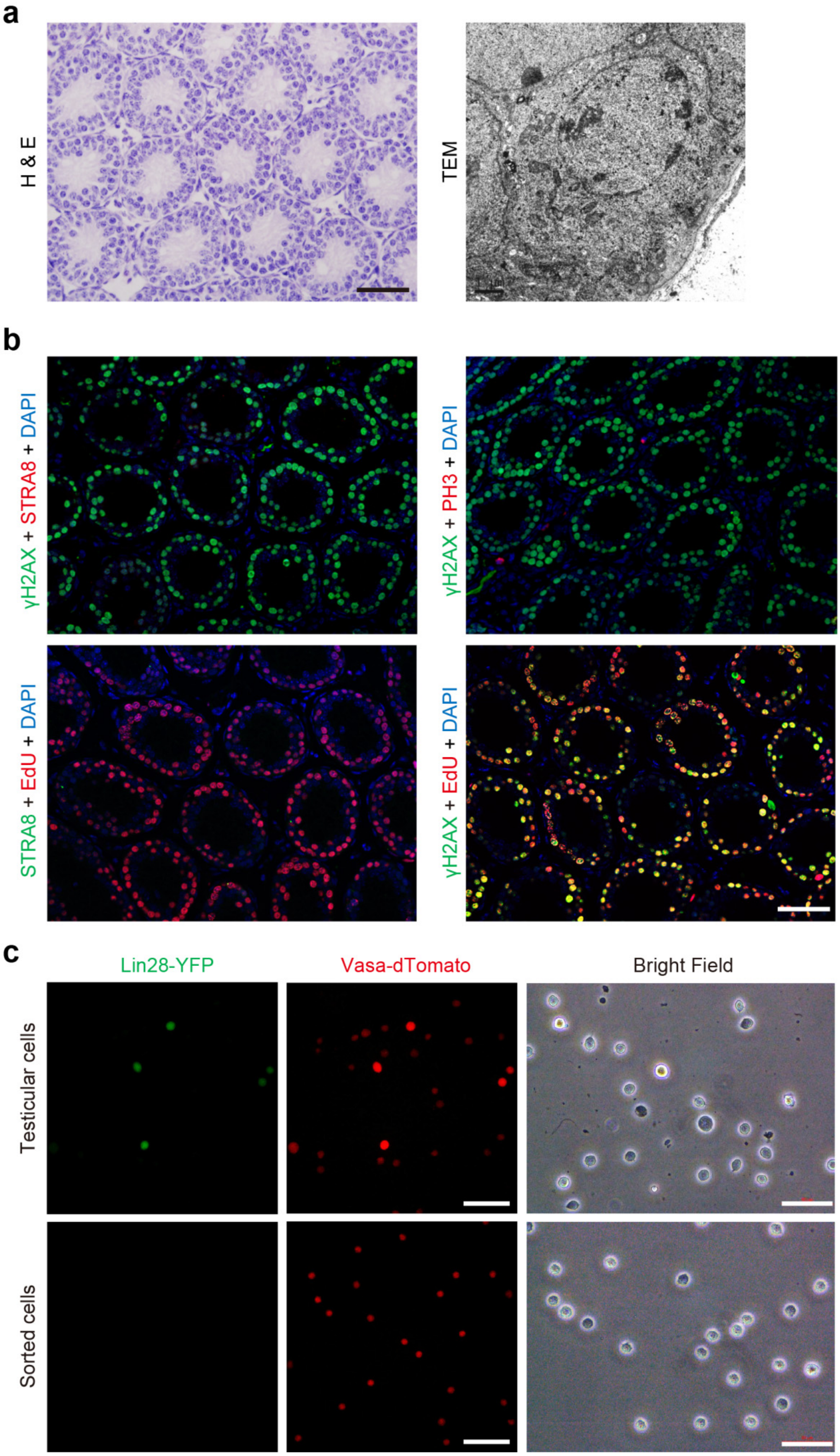

Figure S4 Characterization of S phase of Type B Spermatogonia (BS) in synchronous spermatogenesis. a H&E staining and TEM images depict representative cross sections of testes from mice treated with WIN 18,446 followed by an RA injection and allowed to recover for 130 h. **b** Immunohistochemical staining for γH2AX, STRA8, EdU, and PH3 in sections from mice treated with WIN 18,446/130 h RA. Scale bar, 50 μm. **c** Representative fluorescence images (observed by fluorescence microscope) and bright field images (observed by inverted phase contrast microscope) of total testicular cell population (upper panel) and sorted cell population by FACS (lower panel). Scale bar, 50 μm. The purity of S phase of type B Spermatogonia (BS) is 98.3%.
