# Supplementary material for: Single-cell RNA-seq uncovers dynamic processes and critical regulators in mouse spermatogenesis
Source: Cell Res. 2018 Jul 30;28(9):879–96. doi: 10.1038/s41422-018-0074-y (PMC6123400; doi:10.1038/s41422-018-0074-y)
Supplement: Supplementary file 5 — Supplementary information, Figure S5 [file 41422_2018_74_MOESM5_ESM.pdf]

Supplementary information, Figure S5

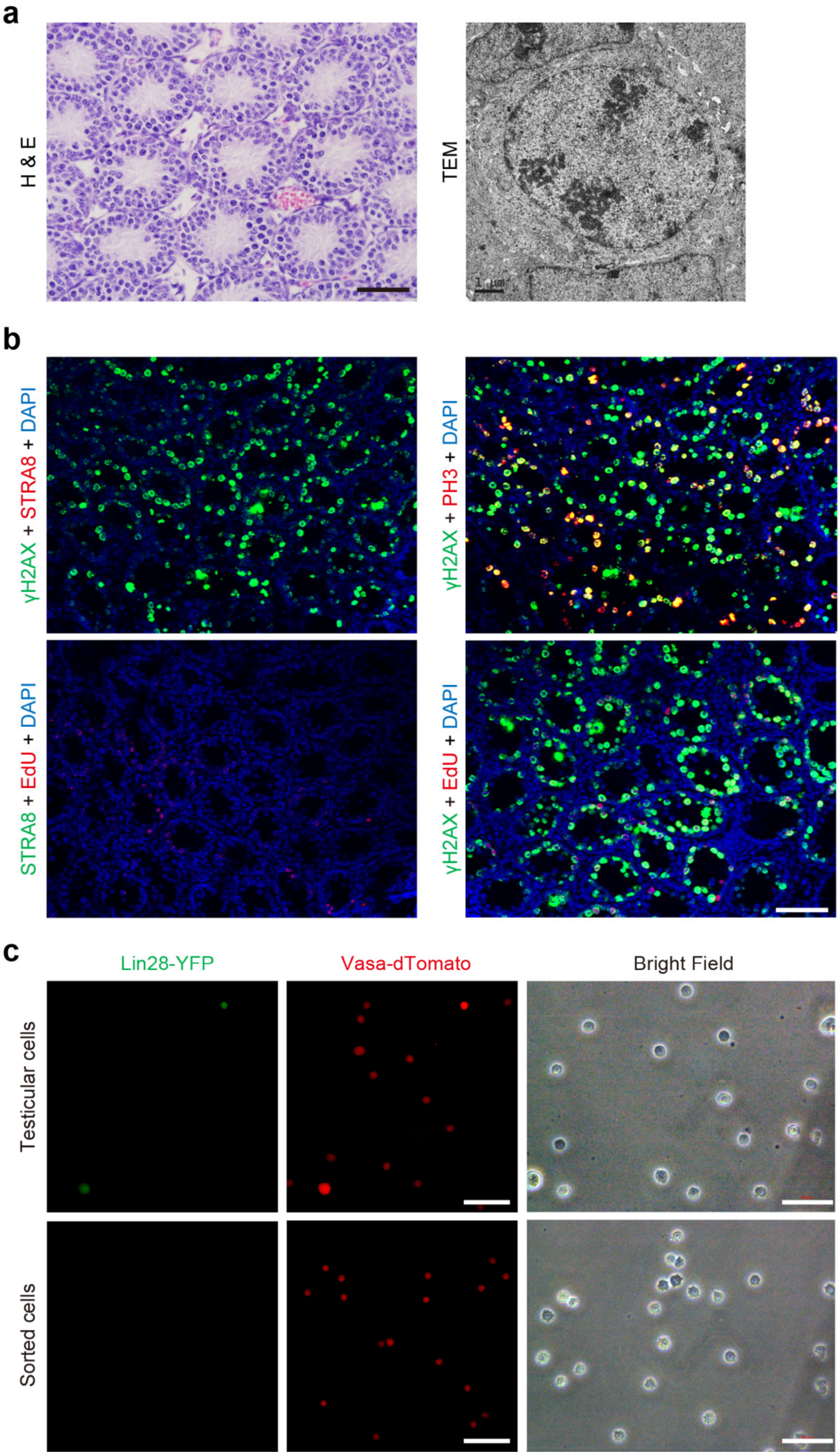

Figure S5 Characterization of G2/M phase of Type B Spermatogonia (BG2) in synchronous spermatogenesis. **a** H&E staining and TEM images depict representative cross sections of testes from mice treated with WIN 18,446 followed by an RA injection and allowed to recover for 140 h. **b** Immunohistochemical staining for γH2AX, STRA8, EdU and PH3 in sections from mice treated with WIN 18,446/140 h RA. **c** Representative fluorescence images (observed by fluorescence microscope) and bright field images (observed by inverted phase contrast microscope) of total testicular cell population (upper panel) and sorted cell population by FACS (lower panel). Scale bar, 50 μm. The purity of G2/M phase of Type B Spermatogonia (BG2) is 92.9%.
