# Supplementary material for: Single-cell RNA-seq uncovers dynamic processes and critical regulators in mouse spermatogenesis
Source: Cell Res. 2018 Jul 30;28(9):879–96. doi: 10.1038/s41422-018-0074-y (PMC6123400; doi:10.1038/s41422-018-0074-y)
Supplement: Supplementary file 7 — Supplementary information, Figure S7 [file 41422_2018_74_MOESM7_ESM.pdf]

Supplementary information, Figure S7

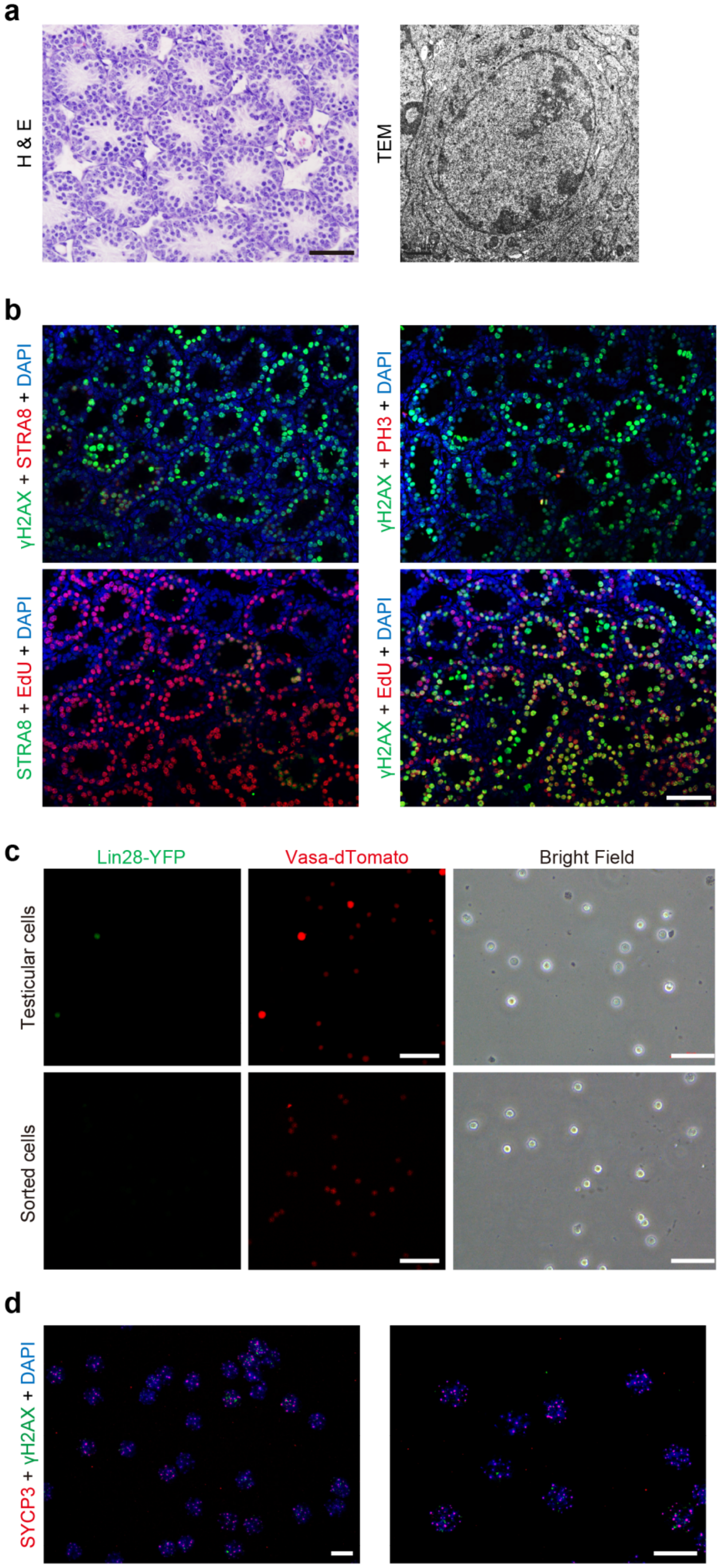

Figure S7 Characterization of early S phase of Preleptotene Spermatocytes (ePL) in synchronous spermatogenesis. **a** H&E staining and TEM images depict representative cross sections of testes from mice treated with WIN 18,446 followed by an RA injection and allowed to recover for 162 h. **b** Immunohistochemical staining for γH2AX, STRA8, EdU, and PH3 in sections from mice treated with WIN 18,446 /162 h RA. Scale bar, 50 μm. **c** Representative fluorescence images (observed by fluorescence microscope) and bright field images (observed by inverted phase contrast microscope) of total testicular cell population (upper panel) and sorted cell population by FACS (lower panel). Scale bar, 50 μm. **d** Surface-spread spermatocyte nuclei immunofluorescence staining for synaptonemal axial element marker SYCP3, γH2AX, and DAPI of the sorted cells. Scale bar, 50 μm. The purity of early S phase of Preleptotene Spermatocytes (ePL) is 97.7%.
