# Supplementary material for: Single-cell RNA-seq uncovers dynamic processes and critical regulators in mouse spermatogenesis
Source: Cell Res. 2018 Jul 30;28(9):879–96. doi: 10.1038/s41422-018-0074-y (PMC6123400; doi:10.1038/s41422-018-0074-y)
Supplement: Supplementary file 11 — Supplementary information, Figure S11 [file 41422_2018_74_MOESM11_ESM.pdf]

Supplementary information, Figure S11

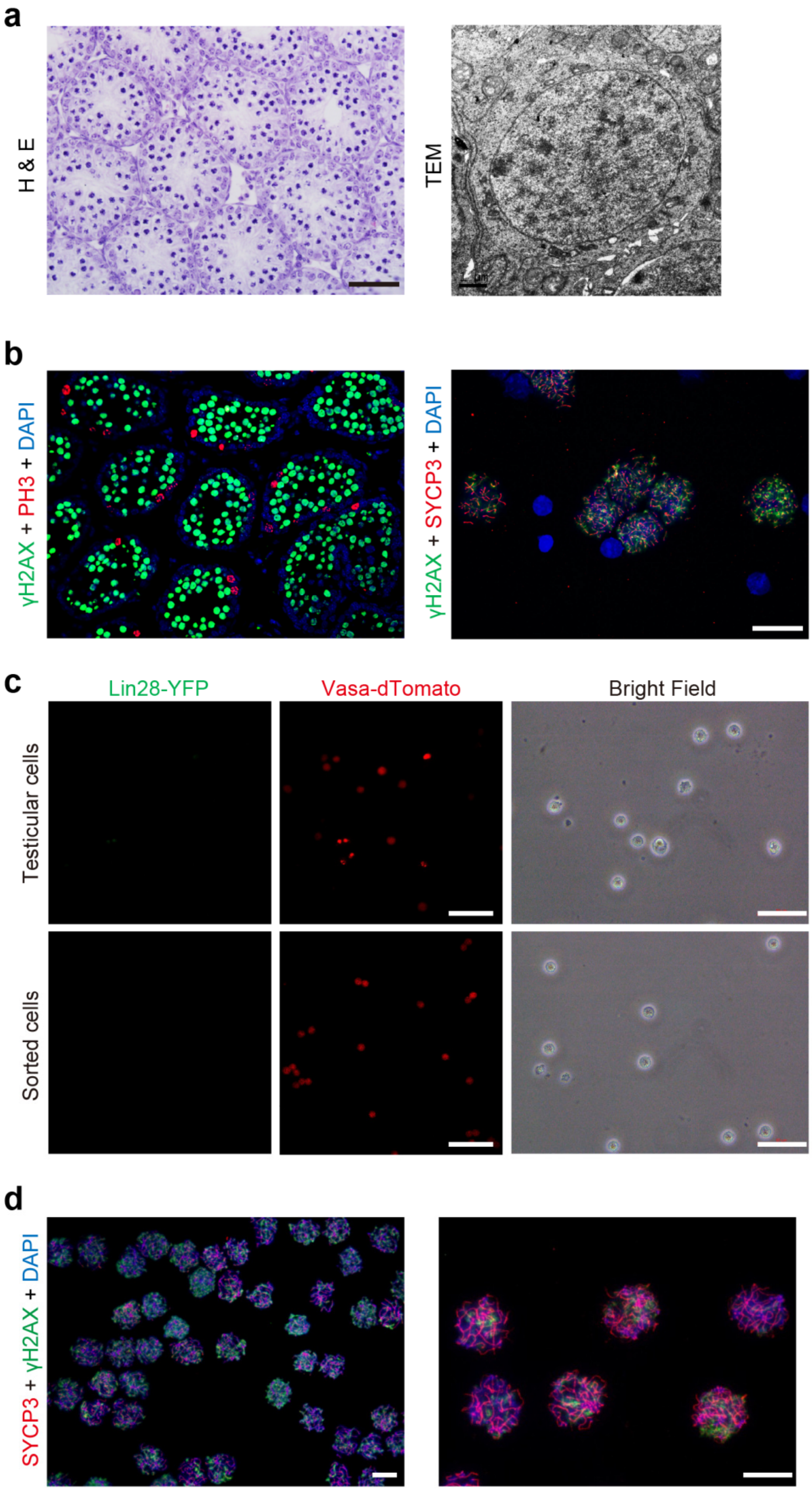

Figure S11 Characterization of Zygotene Spermatocytes (Z) in synchronous spermatogenesis. **a** H&E staining and TEM images depict representative cross sections of testes from mice treated with WIN 18,446 followed by an RA injection and allowed to recover for 216 h. **b** Immunohistochemical staining for γH2AX, and PH3 in sections from mice treated with WIN 18,446/216 h RA. Surface-spread spermatocyte nuclei immunofluorescence staining for SYCP3, γH2AX and DAPI of synchronous total testicular cell population. Scale bar, 50 μm. **c** Representative fluorescence images (observed by fluorescence microscope) and bright field images (observed by inverted phase contrast microscope) of total testicular cell population (upper panel) and sorted cell population by FACS (lower panel). Scale bar, 50 μm. **d** Surface-spread spermatocyte nuclei immunofluorescence staining for SYCP3, γH2AX and DAPI of the sorted cells. Scale bar, 50 μm. The purity of Zygotene Spermatocytes (Z) is 98.5%.
