# Supplementary material for: Single-cell RNA-seq uncovers dynamic processes and critical regulators in mouse spermatogenesis
Source: Cell Res. 2018 Jul 30;28(9):879–96. doi: 10.1038/s41422-018-0074-y (PMC6123400; doi:10.1038/s41422-018-0074-y)
Supplement: Supplementary file 12 — Supplementary information, Figure S12 [file 41422_2018_74_MOESM12_ESM.pdf]

Supplementary information, Figure S12

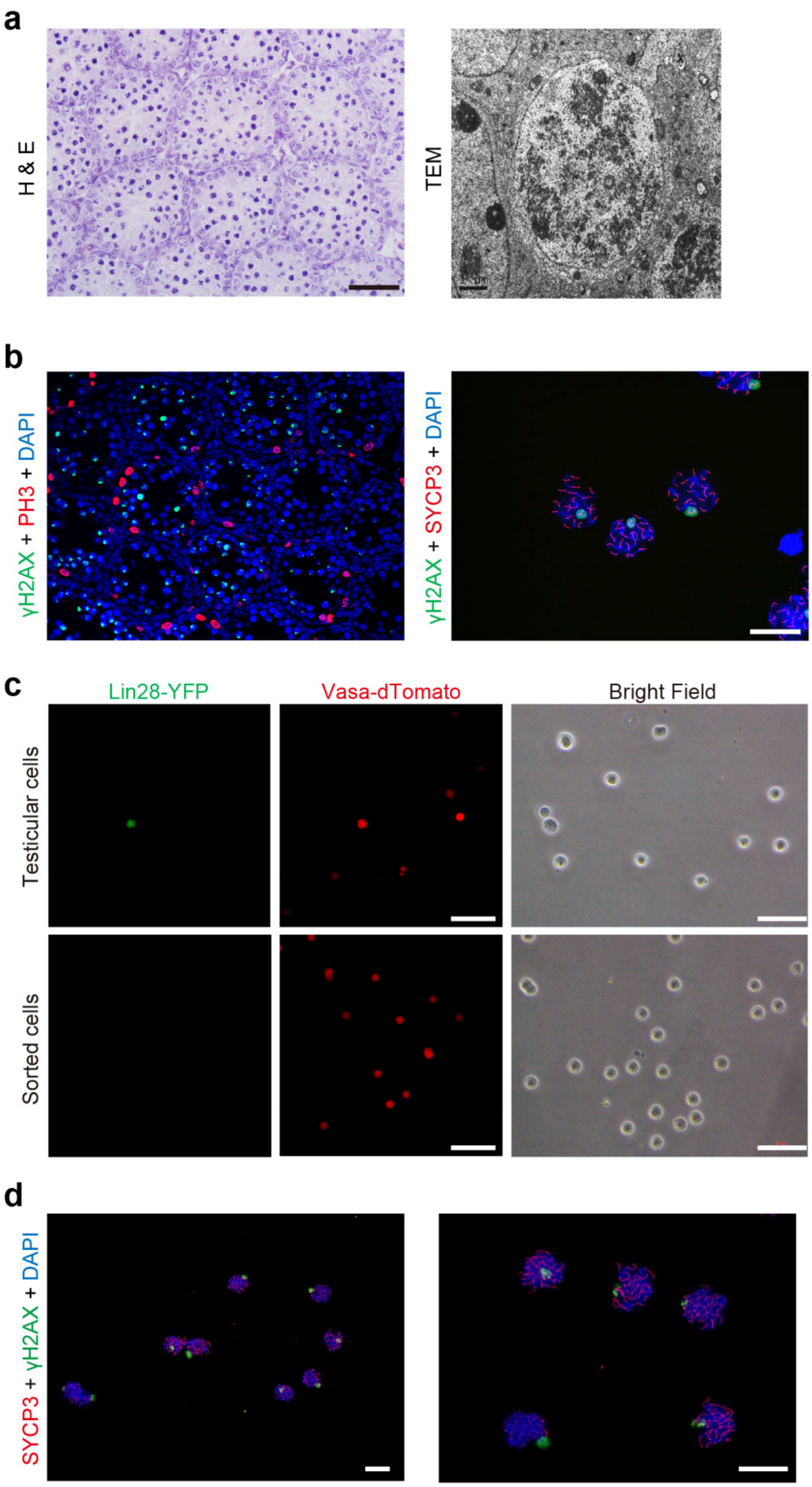

Figure S12 Characterization of early Pachytene Spermatocytes (eP) in synchronous spermatogenesis. a H&E staining and TEM images depict representative cross sections of testes from mice treated with WIN 18,446 followed by an RA injection and allowed to recover for 264 h. **b** Immunohistochemical staining for DSB marker γH2AX, PH3 in sections from mice treated with WIN 18,446/264 h RA. Surface-spread spermatocyte nuclei immunofluorescence staining for SYCP3, γH2AX and DAPI of synchronous total testicular cell population. Scale bar, 50 μm. **c** Representative fluorescence images (observed by fluorescence microscope) and bright field images (observed by inverted phase contrast microscope) of total testicular cell population (upper panel) and sorted cell population by FACS (lower panel). Scale bar, 50 μm. **d** Surface-spread spermatocyte nuclei immunofluorescence staining for SYCP3, γH2AX and DAPI of the sorted cells. Scale bar, 50 μm. The purity of early Pachytene Spermatocytes (eP) is 99.7%.
