# Supplementary material for: Single-cell RNA-seq uncovers dynamic processes and critical regulators in mouse spermatogenesis
Source: Cell Res. 2018 Jul 30;28(9):879–96. doi: 10.1038/s41422-018-0074-y (PMC6123400; doi:10.1038/s41422-018-0074-y)
Supplement: Supplementary file 18 — Supplementary information, Figure S18 [file 41422_2018_74_MOESM18_ESM.pdf]

Supplementary information, Figure S18

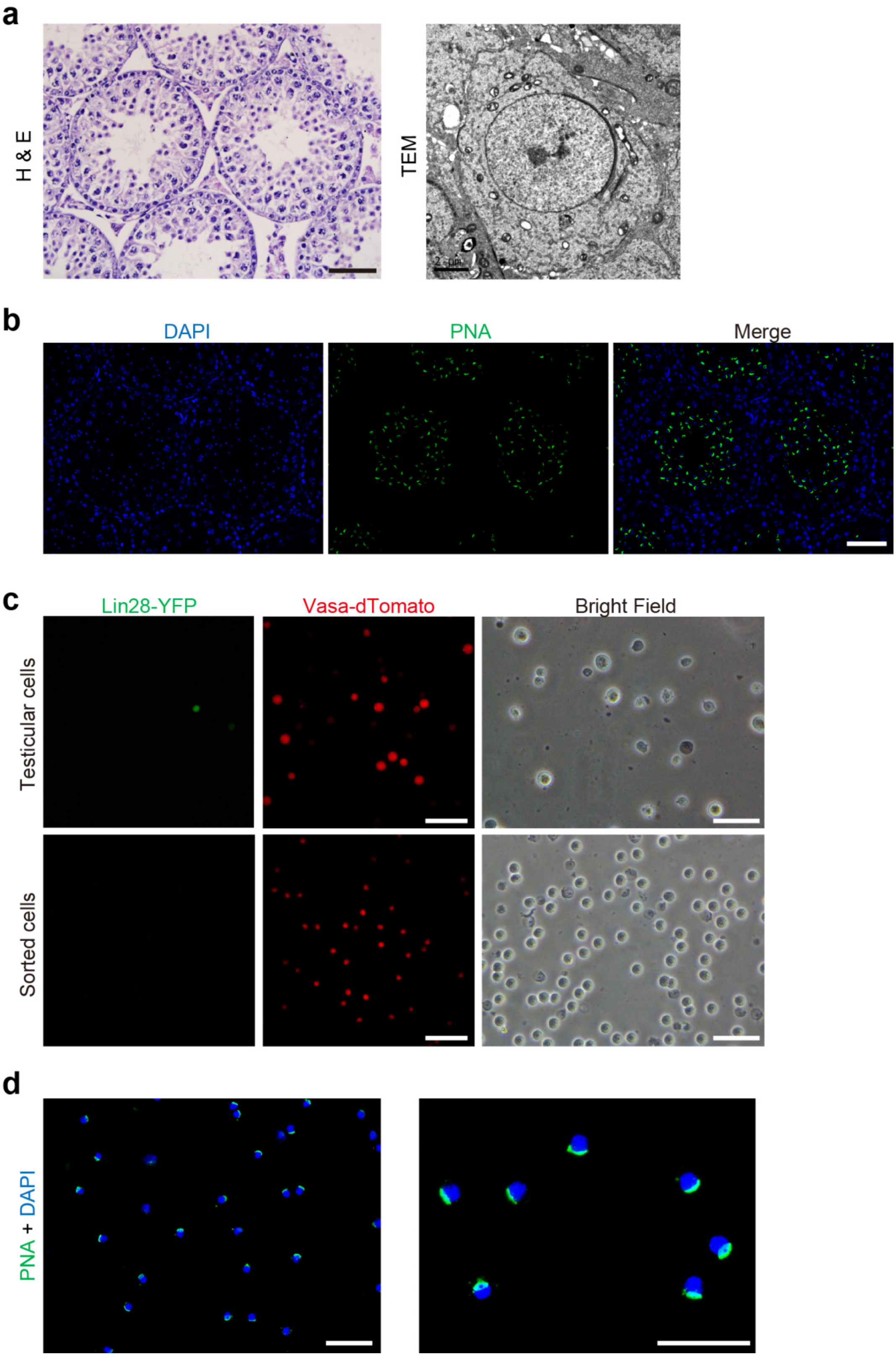

Figure S18 Characterization of Step 5 to Step 6 round Spermatids (RS6) in synchronous spermatogenesis. a H&E staining and TEM images depict representative cross sections of testes from mice treated with WIN 18,446 followed by an RA injection and allowed to recover for 516 h. **b** Immunohistochemical staining for PNA in sections from mice treated with WIN 18,446/516 h RA. Scale bar, 50 μm. **c** Representative fluorescence images (observed by fluorescence microscope) and bright field images (observed by inverted phase contrast microscope) of total testicular cell population (upper panel) and sorted cell population by FACS (lower panel). Scale bar, 50 μm. **d** Immunocytochemical staining for PNA of the sorted cells. Scale bar, 50 μm. The purity of Step 5 to Step 6 round Spermatids (RS6) is 95.4%.
