# Supplementary material for: Single-cell RNA-seq uncovers dynamic processes and critical regulators in mouse spermatogenesis
Source: Cell Res. 2018 Jul 30;28(9):879–96. doi: 10.1038/s41422-018-0074-y (PMC6123400; doi:10.1038/s41422-018-0074-y)
Supplement: Supplementary file 19 — Supplementary information, Figure S19 [file 41422_2018_74_MOESM19_ESM.pdf]

Supplementary information, Figure S19

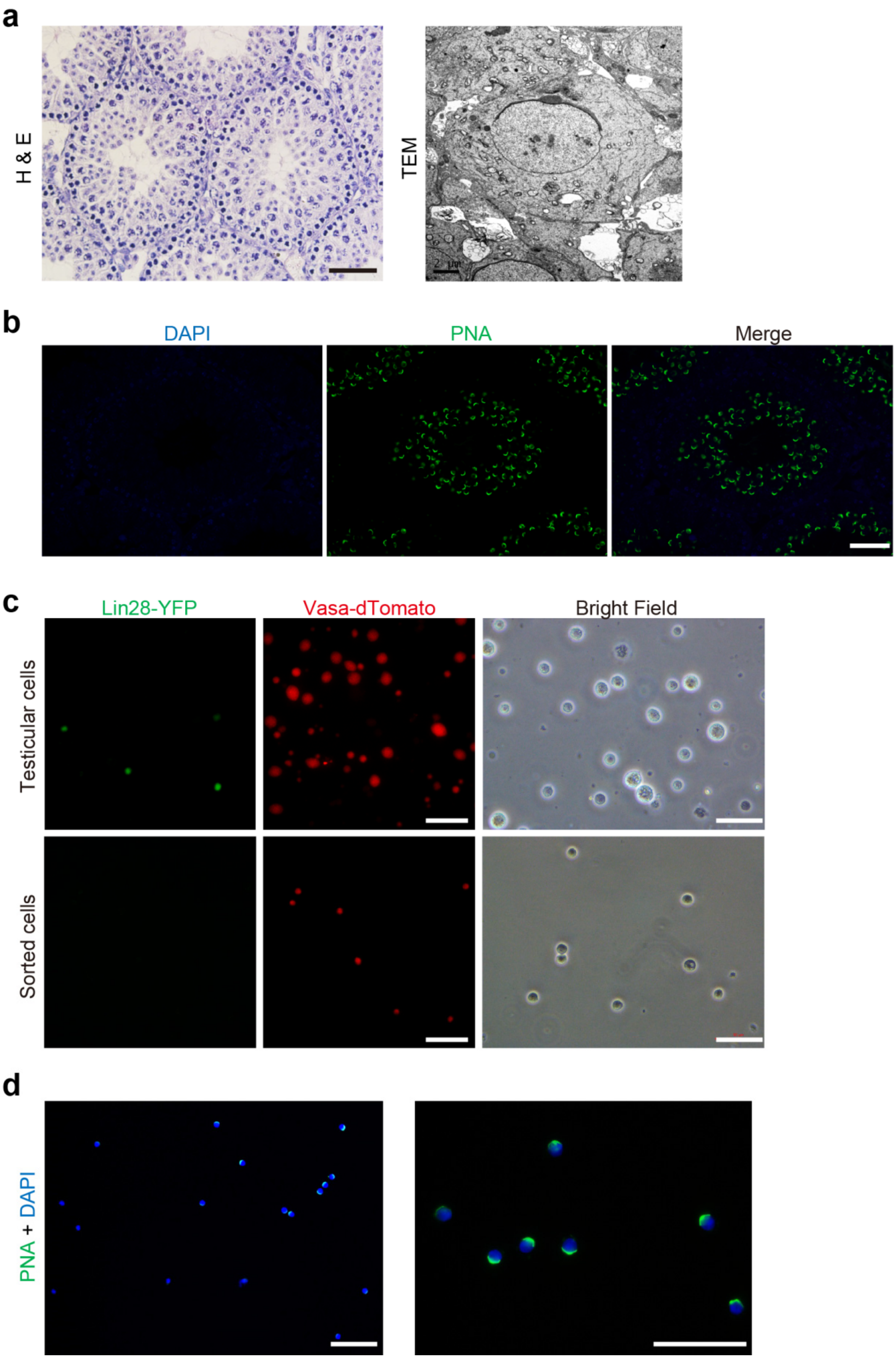

Figure S19 Characterization of Step 7 to Step 8 round Spermatids (RS8) in synchronous spermatogenesis. a H&E staining and TEM images depict representative cross sections of testes from mice treated with WIN 18,446 followed by an RA injection and allowed to recover for 540 h. **b** Immunohistochemical staining for PNA (green) in sections from mice treated with WIN 18,446/540 h RA. Scale bar, 50 μm. **c** Representative fluorescence images (observed by fluorescence microscope) and bright field images (observed by inverted phase contrast microscope) of total testicular cell population (upper panel) and sorted cell population by FACS (lower panel). Scale bar, 50 μm. **d** Immunocytochemical staining for PNA of the sorted cells. Scale bar, 50 μm. The purity of Step 7 to Step 8 round Spermatids (RS8) is 96.7%.
