# Supplementary material for: Single-cell RNA-seq uncovers dynamic processes and critical regulators in mouse spermatogenesis
Source: Cell Res. 2018 Jul 30;28(9):879–96. doi: 10.1038/s41422-018-0074-y (PMC6123400; doi:10.1038/s41422-018-0074-y)
Supplement: Supplementary file 20 — Supplementary information, Figure S20 [file 41422_2018_74_MOESM20_ESM.pdf]

Supplementary information, Figure S20

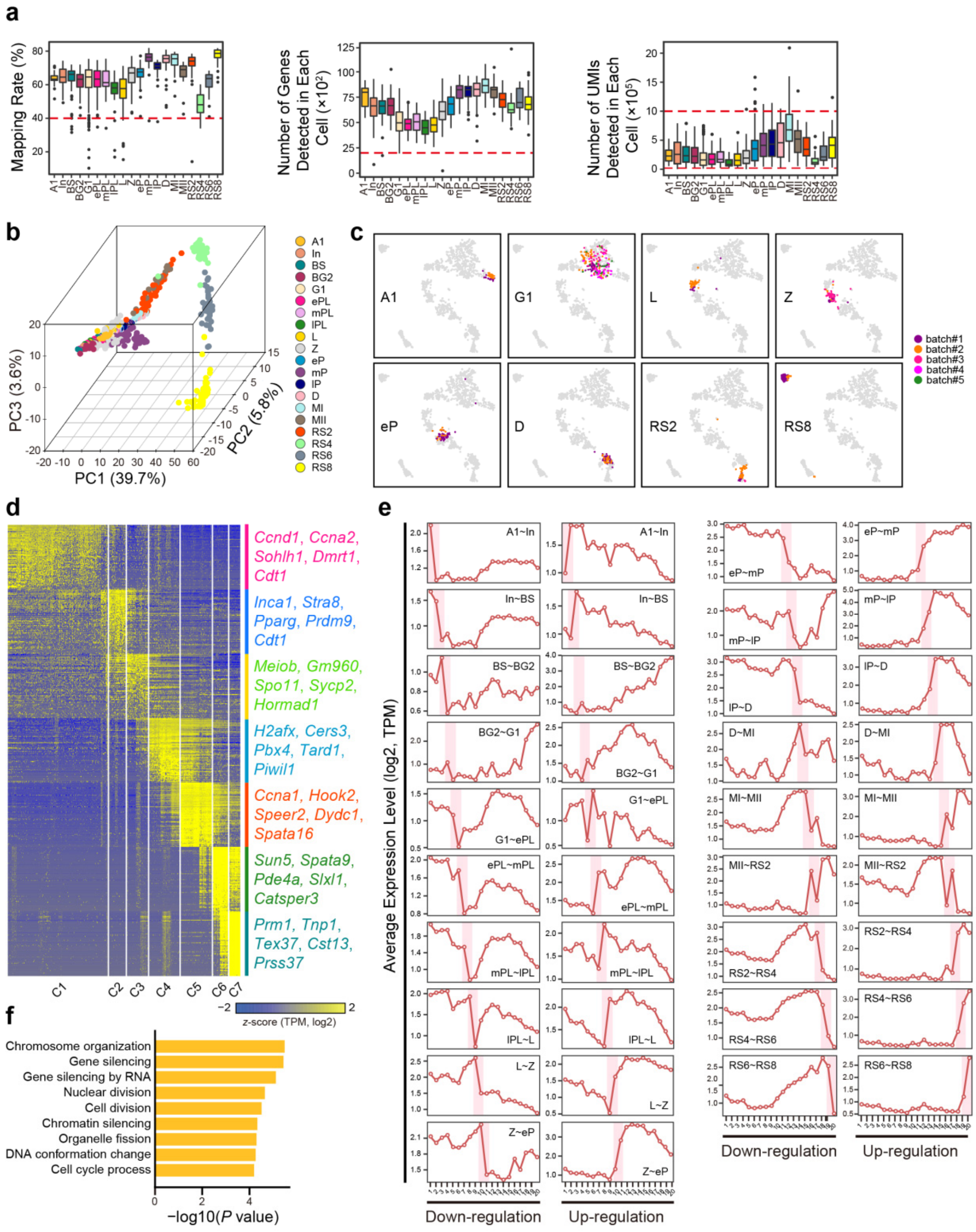

Figure S20 Dynamic gene expression patterns of 1,136 spermatogenic cells. a Quality control information of single-cell RNA-seq. Boxplots showing the mapping rate (left panel), number of genes (middle panel) and number of UMIs detected (right

panel) in each individual cell at 20 developmental stages. The red dash lines represent quality control process standard. **b** Three dimensions PCA plot of spermatogenic cells based on single-cell RNA-seq data. The different developmental stages are presented in distinct colors. **c** *t*-SNE plot showing batch information in representative cell types. The cells in different experimental batches are presented in distinct colors. **d** Heatmap showing normalized expression levels of top 100 DEGs in each individual cell across seven clusters. Top DEGs are selected based on average expression levels. Representative genes are shown on the right. Color key from yellow to blue represents the relative gene expression level from high to low. **e** Line graphs showing the up- and down-regulated DEGs expression levels between two contiguous stages. Expression levels were calculated by $\log_2(TPM/10 + 1)$. *x* axis indicates different developmental stages: 1, A1; 2, In; 3, BS; 4, BG2; 5, G1; 6, ePL; 7, mPL; 8, lPL; 9, L; 10, Z; 11, eP; 12, mP; 13, lP; 14, D; 15, MI; 16, MII; 17, RS2; 18, RS4; 19, RS6; 20, RS8. **f** GO analysis of genes down-regulated from A1 to In.
