# Supplementary material for: Single-cell RNA-seq uncovers dynamic processes and critical regulators in mouse spermatogenesis
Source: Cell Res. 2018 Jul 30;28(9):879–96. doi: 10.1038/s41422-018-0074-y (PMC6123400; doi:10.1038/s41422-018-0074-y)
Supplement: Supplementary file 21 — Supplementary information, Figure S21 [file 41422_2018_74_MOESM21_ESM.pdf]

Supplementary information, Figure S21

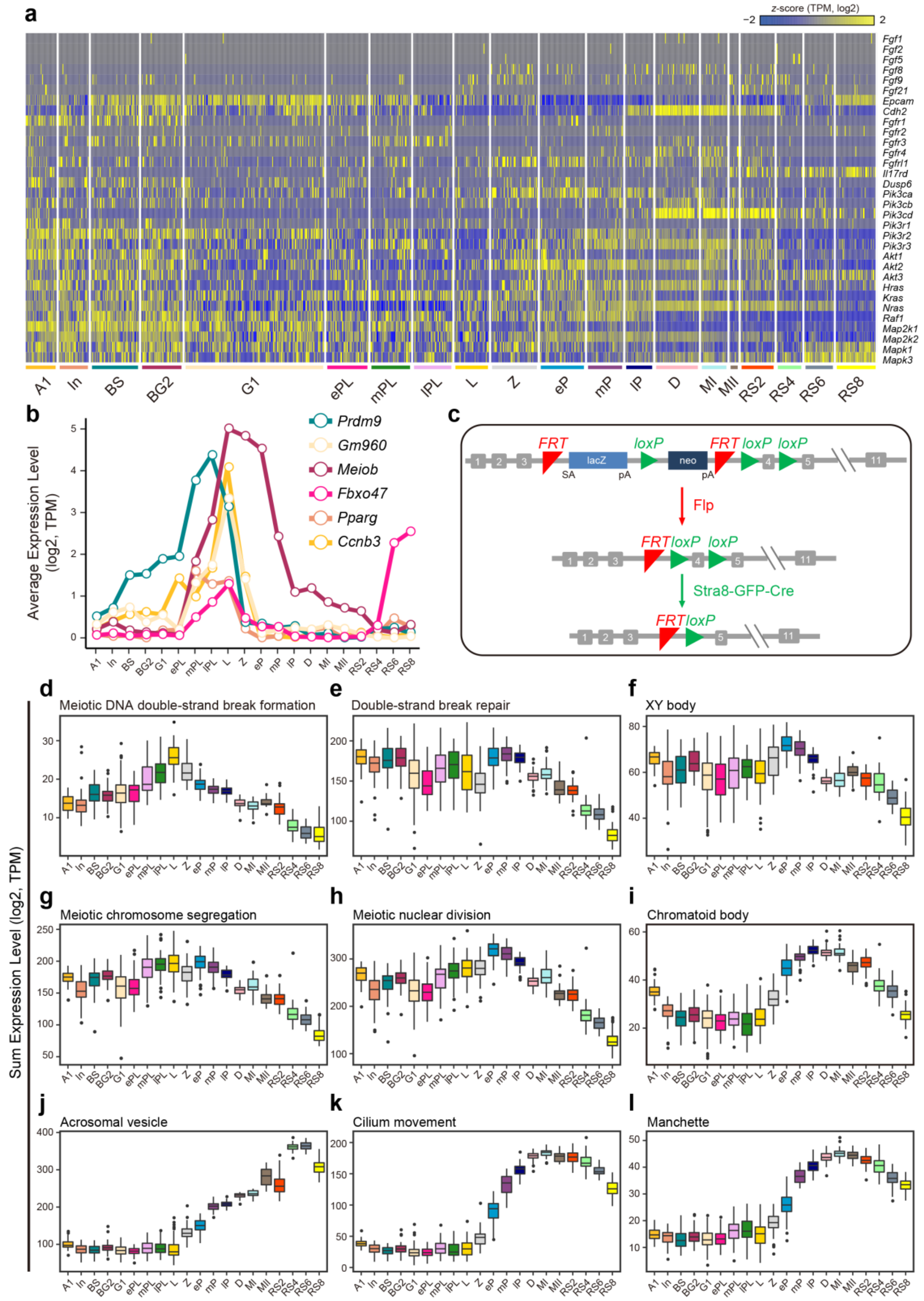

Figure S21 Stage-specifically expressed genes play essential roles in spermatogenesis. a Heatmap showing normalized expression level of key regulators regarding FGF signaling across all stages. The color from yellow to blue indicated the relative expression level detected in each individual cell from high to low. Each row represents a gene as listed on the right. **b** Line plot showing average expression level of previously characterized genes enriched in cluster C3 including *Prdm9*, *Gm960* and *Meiob*, and novel enriched genes including *Fbox47*, *Pparg* and *Ccnb3* across all stages. The circle indicates the mean expression level detected in each individual cell in every developmental stage respectively. Expression levels are transferred to log_2(TPM/10 + 1). **c** Schematic diagram for the "Knockout-first" conditional allele of *Fbxo47*$^{tm1a(EUCOMM)Wtsi}$ mice. **d-l** Boxplots showing gene expression level changes of several representative terms during spermatogenesis. The gene list of meiotic DNA double-strand break formation (**d**), double-strand bread repair via homologous recombination (**e**), XY body (**f**), meiotic chromosome segregation (**g**), meiotic nuclear division (**h**), chromatoid body (**i**), acrosomal vesicle (**j**), cilium movement (**k**) and manchette (**l**) is obtained from MGI (see Materials and Methods). Expression levels are transferred to log_2(TPM/10 + 1).
