# Supplementary material for: Single-cell RNA-seq uncovers dynamic processes and critical regulators in mouse spermatogenesis
Source: Cell Res. 2018 Jul 30;28(9):879–96. doi: 10.1038/s41422-018-0074-y (PMC6123400; doi:10.1038/s41422-018-0074-y)
Supplement: Supplementary file 22 — Supplementary information, Figure S22 [file 41422_2018_74_MOESM22_ESM.pdf]

Supplementary information, Figure S22

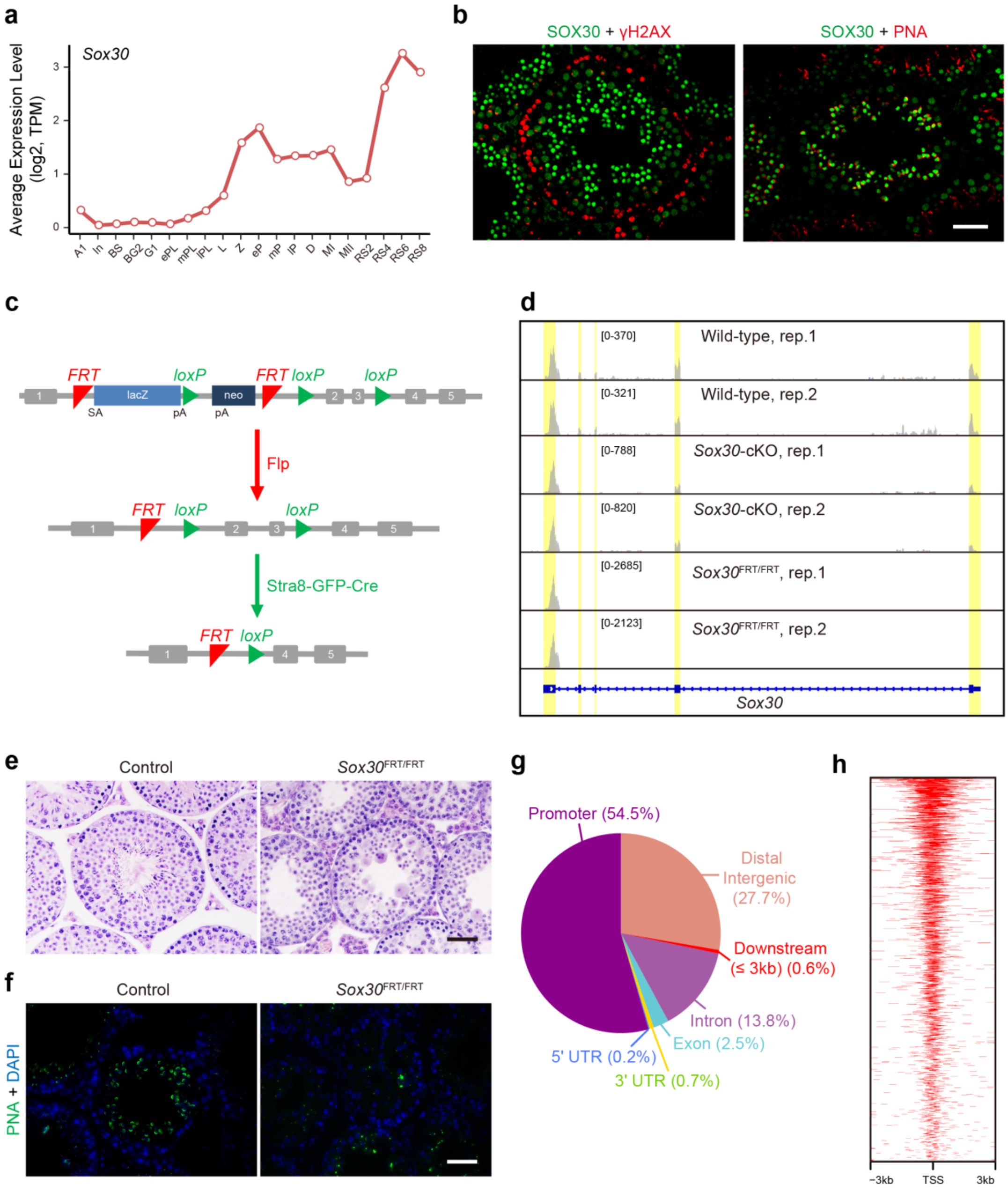

Figure S22 ***Sox30*** **is an essential transcriptional factor for spermiogenesis. a** Line graph showing the average expression level of *Sox30* across all stages in wild-type mice. The circle represents the mean of the expression level, which is calculated by $\log_2(\text{TPM}/10 + 1)$. **b** Immunohistochemical staining for SOX30 (green), γH2AX (red,

left panel), PNA (red, right panel) and DAPI (blue) in sections from adult wild-type mice. Scale bar, 50 μm. **c** Schematic diagram for the "Knockout-first" conditional allele of *Sox30*$^{tm1a(KOMP)Wtsi}$ mice. **d** Snapshops showing the enrichment of RNA-seq reads of *Sox30* in wild-type, *Sox30*-cKO and *Sox30*$^{FRT/FRT}$ by bulk RNA-seq. Each group has two replicates. **e** H&E staining of wild-type control and Sox30$^{FRT/FRT}$ testes sections at 8-weeks old. Scale bar, 50 μm. **f** Testes sections from adult wild-type control and Sox30$^{FRT/FRT}$ mice were immunostained with PNA (green) and DAPI (blue). Scale bar, 50 μm. **g.** Pie chart showing the distribution of peaks in genomic elements called from *Sox30* wild-type mice by bulk ChIP-seq. The total number of peaks is 2,959. The number in the parentheses indicates the percentages of peaks belonging to different genomic regions. **h** Heatmap showing the peak enrichment of *Sox30* wild-type mice in promoter regions by bulk ChIP-seq. The promoter is defined as transcription start site (TSS) ± 3 kb. Each row represents a promoter region of a gene. The intensity from red to white stands for the relative enrichment from high to low.
