# Supplementary material for: Single-cell RNA-seq uncovers dynamic processes and critical regulators in mouse spermatogenesis
Source: Cell Res. 2018 Jul 30;28(9):879–96. doi: 10.1038/s41422-018-0074-y (PMC6123400; doi:10.1038/s41422-018-0074-y)
Supplement: Supplementary file 23 — Supplementary information, Figure S23 [file 41422_2018_74_MOESM23_ESM.pdf]

Supplementary information, Figure S23

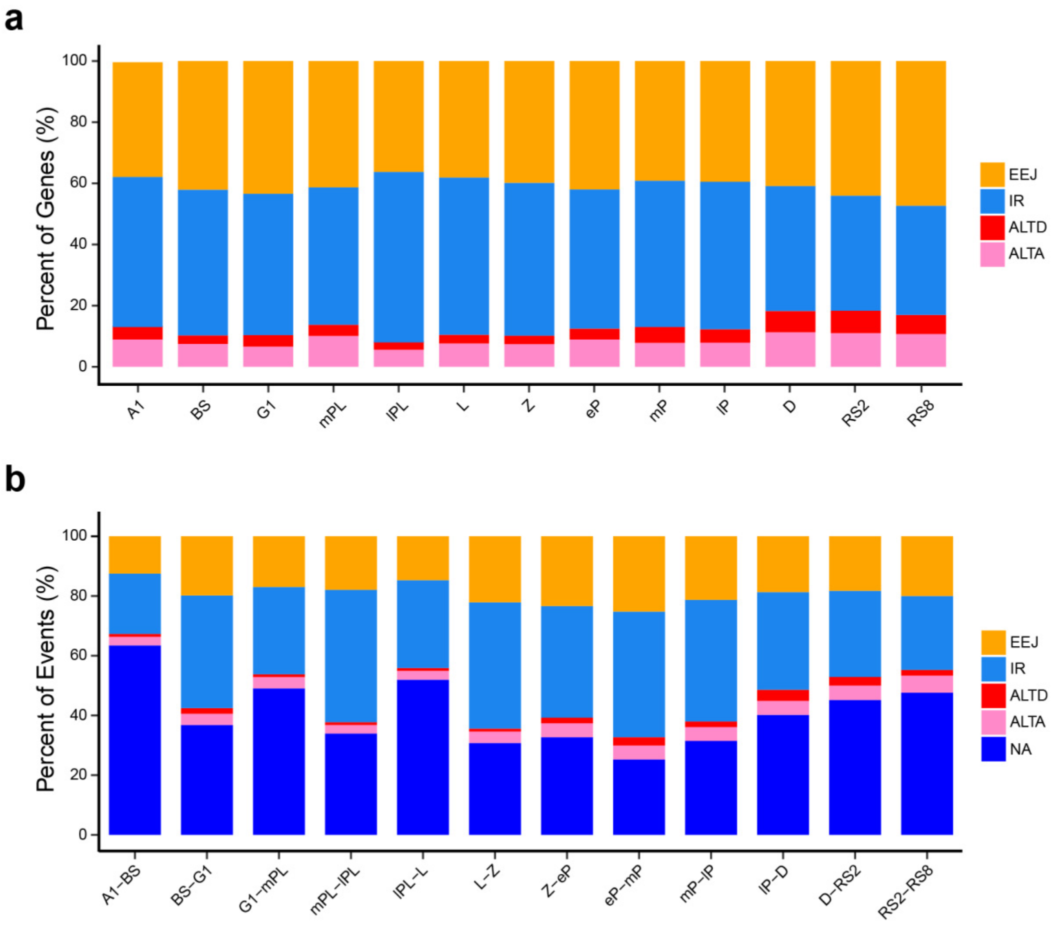

Figure S23 Alternative splicing events during spermatogenesis. a Stacked barplot showing the percentage of genes affected by four types of alternative splicing (AS) events detected at each stage. These genes were affected by at least two AS events of the same type in each stage. EEJ, exon-exon junction; IR, intron retention; ALTD, alternative donors; ALTA, alternative acceptors. **b** Stacked barplot showing the percentage of AS events changing from the previous stage to the next stage. When we compared two consecutive stages, the AS type of a gene is classified by its status in the latter stage.
