# Supplementary material for: Single-cell RNA-seq uncovers dynamic processes and critical regulators in mouse spermatogenesis
Source: Cell Res. 2018 Jul 30;28(9):879–96. doi: 10.1038/s41422-018-0074-y (PMC6123400; doi:10.1038/s41422-018-0074-y)
Supplement: Supplementary file 24 — Supplementary information, Figure S24 [file 41422_2018_74_MOESM24_ESM.pdf]

Supplementary information, Figure S24

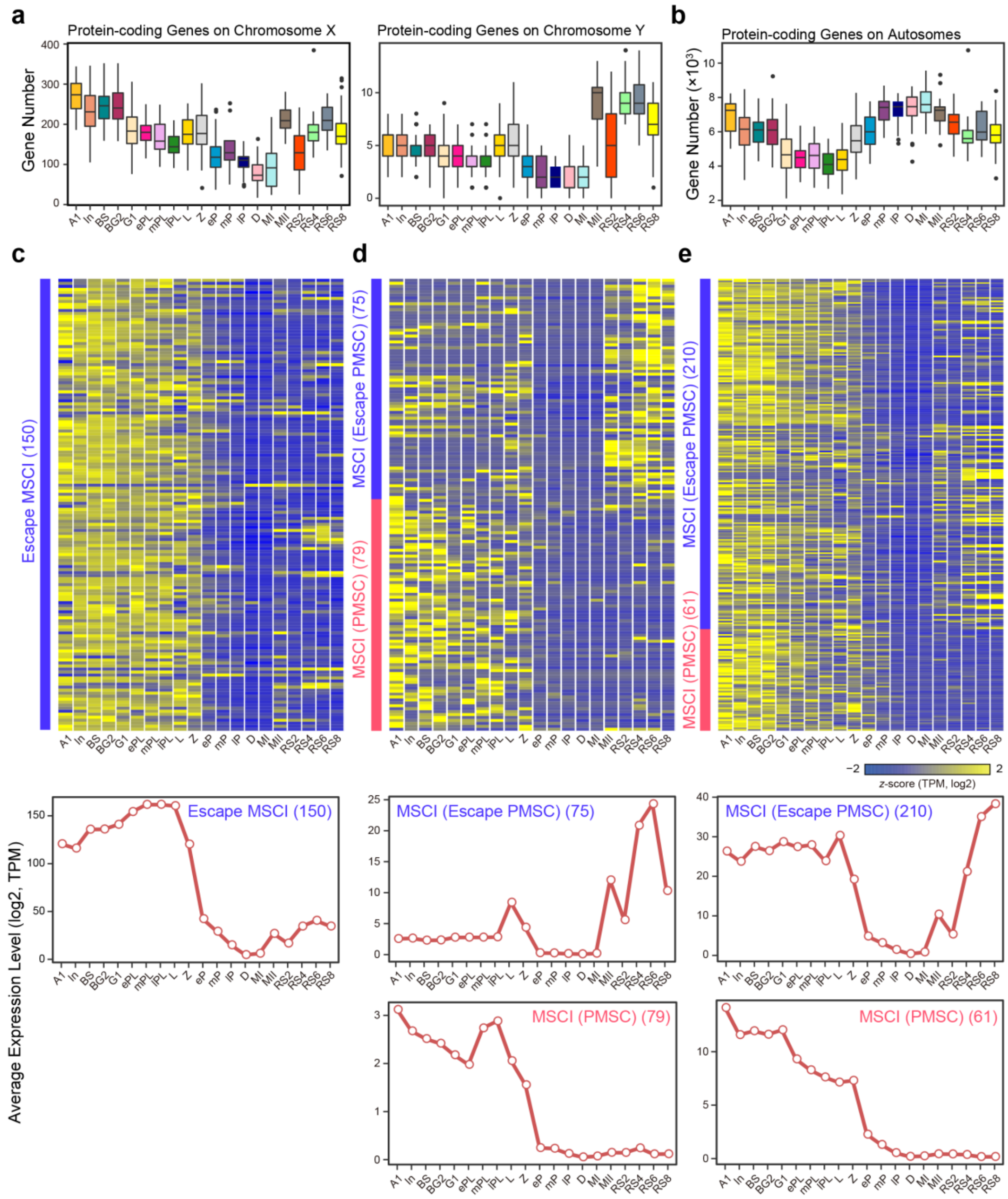

Figure S24 Sex-linked protein-coding gene expression analysis during spermatogenesis. a Boxplots showing the number of expressed protein-coding genes in chromosome X (left panel) and chromosome Y (right panel) detected in each cell at 20 developmental stages. **b** Boxplots showing the number of expressed protein-coding genes in autosomes detected in each individual cell at 20 developmental stages. **c-e**

Heatmaps showing MSCI Escape (**c**), type I (**d**) and non-type I (**e**) gene expression patterns. Row color bar representing genes of MSCI (PMSC) (pink) and MSCI (Escape PMSC) (blue), respectively. The number in parentheses indicates the gene number of each event. Line plots in the bottom panels show average gene expression levels in these three gene types. The circles represent the mean gene expression levels. Expression levels are transferred to $\log_2(\mathrm{TPM}/10 + 1)$.
